# Scalable Electrocatalytic Urea Wastewater Treatment Coupled with Hydrogen Production by Regulating Adsorption Behavior of Urea Molecule

**DOI:** 10.1007/s40820-024-01585-0

**Published:** 2025-02-24

**Authors:** Chunming Yang, Huijuan Pang, Xiang Li, Xueyan Zheng, Tingting Wei, Xu Ma, Qi Wang, Chuantao Wang, Danjun Wang, Bin Xu

**Affiliations:** 1 Shaanxi Key Laboratory of Chemical Reaction Engineering, School of Chemistry and Chemical Engineering, Yan’an University, Yan’an, 716000 People’s Republic of China; 2 College of Materials Science and Engineering, Beijing University of Chemical Technology, Beijing, 100029 People’s Republic of China; 3 Hubei Three Gorges Laboratory, Yichang, 443007 People’s Republic of China

**Keywords:** Urea wastewater treatment, Hydrogen production, Adsorption behavior, Heterogeneous interface

## Abstract

Electrocatalytic urea wastewater treatment technology has emerged as a promising method for environmental remediation. However, the realization of highly efficient and scalable electrocatalytic urea wastewater treatment (SEUWT) is still an enormous challenge. Herein, through regulating the adsorption behavior of urea functional groups, the efficient SEUWT coupled hydrogen production is realized in anion exchange membrane water electrolyzer (AEMWE). Density functional theory calculations indicate that self-driven electron transfer at the heterogeneous interface (NiO/Co_3_O_4_) can induce charge redistribution, resulting in electron-rich NiO and electron-deficient Co_3_O_4_, which are superior to adsorbing C=O (electron-withdrawing group) and –NH_2_ (electron-donating group), respectively, regulating the adsorption behavior of urea molecule and accelerating the reaction kinetics of urea oxidation. This viewpoint is further verified by temperature-programmed desorption experiments. The SEUWT coupled hydrogen production in AEMWE assembled with NiO/Co_3_O_4_ (anode) and NiCoP (cathode) can continuously treat urea wastewater at an initial current density of 600 mA cm^−2^, with the average urea treatment efficiency about 53%. Compared with overall water splitting, the H_2_ production rate (8.33 mmol s^−1^) increases by approximately 3.5 times. This work provides a cost-effective strategy for scalable purifying urea-rich wastewater and energy-saving hydrogen production.

**Supplementary Information:**

The online version contains supplementary material available at 10.1007/s40820-024-01585-0.

## Highlights

- The heterogeneous interface of NiO/Co_3_O_4_ was constructed for regulating adsorption behavior of urea functional groups.
- The regulation mechanism of urea molecular adsorption behavior was verified by temperature-programmed desorption experiments and density functional theory calculations.
- Efficient and scalable electrocatalytic urea wastewater treatment coupled with hydrogen production was realized in anion exchange membrane water electrolyzer AEMWE, which can continuously treat urea wastewater at an initial current density of 600 mA cm^-2^, with about 53% in average urea treatment efficiency.

**Supplementary Information:**

The online version contains supplementary material available at 10.1007/s40820-024-01585-0.

## Graphical abstract

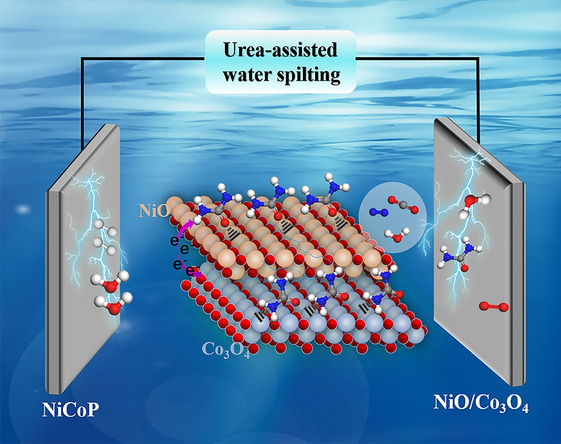

## Introduction

Urea is a vital active nitrogen compound in the nitrogen cycle, playing a crucial role in water, energy and food domains [1, 2]. The large quantities of urea-rich domestic and industrial wastewater without treatment will produce harmful toxins, threatening water ecological balance [3, 4]. Traditional urea decomposition methods, such as enzymatic hydrolysis, biomass degradation and chemical oxidation, often have low economic benefits, complex technology and harsh working environments, making it difficult to meet industrial requirements [5]. It is important to note that electrochemical urea oxidation reaction (CO(NH_2_)_2_ + 6OH^−^  → N_2_ + 5H_2_O + CO_2_ + 6e^−^, UOR) is considered to be a cost-effective method for treating urea-rich wastewater [6]. In particular, UOR has a wide range of applications in energy conversion and storage, including urea-assisted hydrogen production, direct urea fuel cells, photoelectrochemical urea decomposition and wastewater treatment [1, 7]. Ideally, urea-assisted hydrogen production, combined with urea wastewater treatment, can achieve purification of urea-rich wastewater and energy-saving hydrogen production [8, 9]. At present, research on this strategy was limited to laboratory scale, and the realization of highly efficient and scalable electrocatalytic urea wastewater treatment (SEUWT) is still an enormous challenge under industrial current densities in water electrolysis equipment such as anion exchange membrane water electrolyzer (AEMWE) [10, 11].

Urea molecule contains two electron-donating groups (–NH_2_) and one electron-withdrawing group (C=O), which tend to adsorb in the electron-deficient and electron-rich regions of the catalyst, respectively [12, 13]. Due to the influence of functional groups in urea on adsorption behavior, it is of great significance to reveal the regulation mechanism of urea adsorption behavior and search for suitable catalysts for UOR [13]. The investigation of semiconductor physics suggests that by utilizing two semiconductors with different energy structures, it is possible to construct a heterojunction, where an internal electric field and two opposing charge distribution regions can be formed at the heterojunction interface [14–16]. Transition metal-based (such as Ni, Co and Fe metals) sulfides, selenides and nitrides are highly efficient catalysts for UOR [17]. However, the strong polarization and fast reaction rate at high current density can lead to the dissolution of nonmetallic elements, affecting the stability of the system [18, 19]. Inversely, heterojunctions formed by transition metal oxides (TMOs) are considered potential catalysts for UOR due to their simple synthesis, stable structure and suitability for high currents [20, 21].

Herein, metal organic framework (MOF) derivatives NiO/Co_3_O_4_ were constructed using a hydrothermal–calcination method for SEUWT coupled hydrogen production. Density functional theory (DFT) results show that the work functions of NiO and Co_3_O_4_ are 8.08 and 6.71 eV, respectively. An internal electric field formed at NiO/Co_3_O_4_ interface caused electrons to transfer from Co_3_O_4_ to NiO and result in the electron-deficient Co_3_O_4_ and electron-rich NiO. The UOR reaction mechanism was revealed through experiment results and temperature-programmed desorption (TPD). The synergistic effect of NiO/Co_3_O_4_ heterojunction interface enhances the adsorption capacity of the catalyst for –NH_2_ (electron-donating group) and C=O (electron-withdrawing group), respectively, regulating the overall adsorption behavior of urea and exhibiting excellent reaction kinetics for UOR. In AEMWE, the SEUWT coupled hydrogen production (UOR||HER) was constructed using NiO/Co_3_O_4_ and NiCoP as anode and cathode, respectively. Compared to overall water splitting, the UOR||HER system can continuously treat urea wastewater at an initial current density of 600 mA cm^−2^, with about 53% in average urea treatment efficiency and approximately 3.5-fold H_2_ yield.

## Experimental Section

### Materials

Nickel chloride hexahydrate (NiCl_2_·6H_2_O) and terephthalic acid (C_8_H_6_O_4_, TPA) were purchased from Beijing Innochem Technology Co., Ltd. Cobalt chloride hexahydrate (CoCl_2_·6H_2_O) and N–N dimethylformamide (DMF) were purchased from Shanghai Aladdin Biochemical Technology Co., Ltd. Nickel foam (NF) was purchased from Tianjin Annuohe New Energy Technology Co., Ltd. Urea (CH_4_N_2_O) and sodium hypophosphite monohydrate (NaH_2_PO_2_·H_2_O) were bought from Shanghai McLean Biochemical Technology Co., Ltd. Absolute ethyl alcohol (C_2_H_5_OH) was purchased form Tianjing YongSheng Fine Chemical Co., Ltd. Deionized water (resistivity > 18.2 MΩ cm) was used to prepare all solutions, and all the chemicals were used as received without treatment.

### Synthesis of NiCo MOF

NiCo MOF precursor was synthesized on NF by a hydrothermal method. Firstly, 0.3 mmol NiCl_2_·6H_2_O, 0.3 mmol CoCl_2_·6H_2_O and 1 mmol TPA were dissolved in 35 mL solution composed of 2.5 mL ethanol, 2.5 mL water and 30 mL DMF. The mixture was stirred in the reactor for 30 min until all the reagents were dissolved. Subsequently, the prepared NF (sonicate in 3 M HCl for 15 min) was transferred into the above solution. After 6 h of hydrothermal treatment at 125 °C and cooling to room temperature, the sample was washed several times with C_2_H_5_OH and deionized water, and then dried to obtain the precursor of NiCo MOF. Under the same conditions, Ni MOF and Co MOF precursors were prepared by changing the molar feed ratio of the reagent. Here, Co MOF was prepared as powder.

### Synthesis of NiO/Co_3_O_4_

NiCo MOF, Ni MOF and Co MOF precursors were heated at a rate of 5 °C min^−1^ in a muffle furnace to 350 °C calcined for 2 h and then dropped to room temperature to obtain NiO/Co_3_O_4_, NiO and Co_3_O_4_.

### Synthesis of NiCoP

In a tubular furnace, 0.5 g NaH_2_PO_2_·H_2_O was uniformly dispersed as a P-source at the bottom of the porcelain boat, and the NiCo MOF was placed above another porcelain vessel, locating them upstream and downstream, respectively. In argon atmosphere, heating at a rate of 5 °C min^−1^ to 350 °C and maintain at 350 ℃ for 2 h. After cooling to room temperature, the obtained sample was washed several times with C_2_H_5_OH and deionized water, and dried to obtain NiCoP.

### Material Characterization

The crystal structure was obtained by X-ray diffraction (XRD, 700SHIMADZU) with Cu Kα radiation source. The morphology was characterized by field emission scanning electron microscopy (SEM, JSM-7610F), transmission electron microscopy (TEM, JEOL F200) and energy-dispersive X-ray spectroscopy (EDS). The elemental composition of samples was analyzed using X-ray photoelectron spectroscopy (XPS, PHI-5000CESCA, Al Kα source and *hν* = 1253.6 eV). All the binding energies were calibrated by using C 1*s* spectrum at 284.8 eV. Normal Raman spectroscopy (*λ* = 532 nm) is obtained on HORIBA (France) without infrared correction. The ultraviolet–visible (UV–Vis) absorbance spectra were measured by spectrophotometer (Beijing Purkinje General T6 new century) for measuring the absorbance of the sample. The UV–Vis diffuse reflectance spectrum was collected on a UV spectrophotometer (Shimadzu, UV-2000) for studying optical properties.

### Electrochemical Characterization

All electrochemical tests were performed on Koster workstations (CS301M) using a standard three-electrode system with the prepared catalyst (1 × 1 cm^2^) as working electrode, graphite rod as counter electrode and Hg/HgO as reference electrode. All high-current data tests are conducted in AEMWE. All experimental data for hydrogen evolution reactions (HER) and oxygen evolution reaction (OER) were tested in 1 M KOH electrolyte solution, while all UOR data were tested in 1 M KOH containing 0.33 M urea. Linear sweep voltammetry (LSV) was tested at a scan rate of 5 mV s^−1^. Electrochemical impedance spectroscopy (EIS) measurements were taken in a frequency range of 100 kHz to 0.1 kHz. The stability of the catalyst was tested by chronopotentiometric (*i–t*), frequency sweep–potentiostat mode, frequency sweep–constant current mode and continuous cyclic voltammetry (CV) method. In the scanning rate range of 10–100 mV s^−1^, electrochemical double-layer capacitance (*C*_dl_) was evaluated by CV curve, which CV curve was linearly proportional to the electrochemically active surface area (ECSA).

### AEMWE Experiment

AEMWE is mainly composed of six parts: anode plate, anode electrode NiO/Co_3_O_4_ (3 × 3 cm^2^), anion exchange membrane, cathode electrode NiCoP (3 × 3 cm^2^), cathode plate and sealing gasket. NiO/Co_3_O_4_ was used for anodic urea oxidation (electrolyte solution with 0.33 M urea of 600 mL), and NiCoP was used for cathodic hydrogen evolution (electrolyte solution with 1 M KOH of 600 mL). The urea oxidation coupled hydrogen evolution properties of NiO/Co_3_O_4_||NiCoP were tested at room temperature in the voltage range of 1–3 V.

### Theoretical Calculations

All calculations were conducted using the Vienna Computational Simulation Package (VASP) based on DFT. The differential charge density calculations are based on the generalized gradient (GGA) of PBE functional and the basis set of flat wave expansion, with a cutoff energy of 450 eV. The Brillouin zone integration was conducted by using a 4 × 4 × 1 k-point mesh for structural relaxation and free energy calculation. The accompanying material includes calculation information.

## Results and Discussion

### Synthesis and Characterization of NiO/Co_3_O_4_

**Fig. 1:**
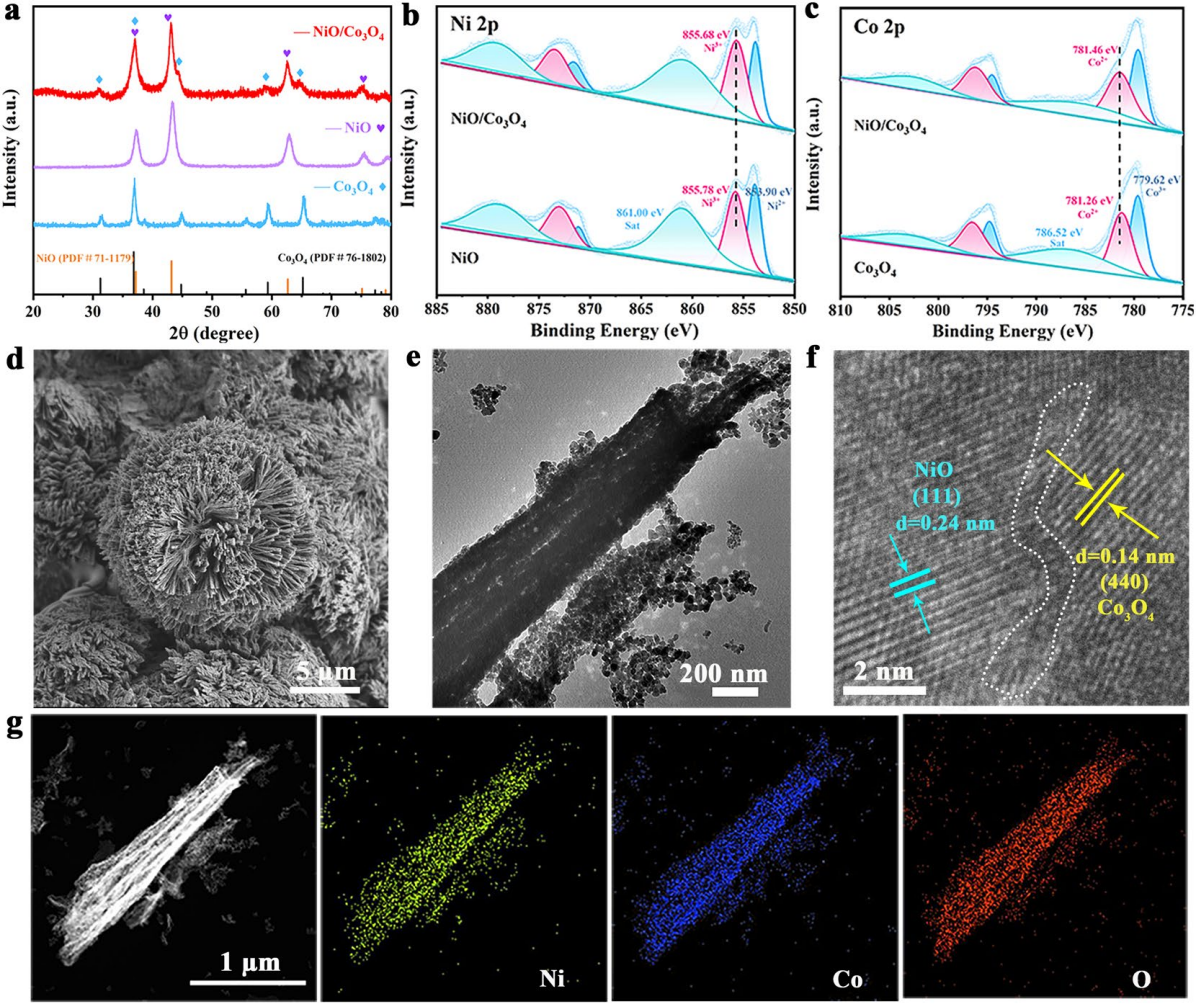
**a** XRD patterns of Co_3_O_4_, NiO and NiO/Co_3_O_4_. **b** High-resolution XPS spectrums of Ni 2*p*, **c** Co 2*p*, **d** SEM image, **e** TEM image, **f** HRTEM image and **g** corresponding elemental mapping images of NiO/Co_3_O_4_

The catalytic performance of different MOF precursors and their oxide is shown in Fig. S1. Although MOF precursors have a relatively low initial potential, their oxide have better performance at high potentials (1.6–1.8 V_RHE_), so we choose them for the further study. The synthesis schematic diagram of NiO/Co_3_O_4_ is shown in Fig. S2. NiO/Co_3_O_4_ was prepared by a hydrothermal–calcination method, using NF as a scaffold template. As shown in Fig. 1a, XRD diagram indicates that all diffraction peaks can be clearly indexed to the standard cards of NiO (PDF#71-1179) and Co_3_O_4_ (PDF#76-1802). The XPS survey spectra of NiO, Co_3_O_4_ and NiO/Co_3_O_4_ are shown in Fig. S3. In regard to the high-resolution Ni 2*p* spectrum of NiO (Fig. 1b), the peaks observed at 853.90 and 871.14 eV are attributed to Ni^2+^. Meanwhile, the peaks observed at 855.78 and 872.99 eV belong to Ni^3+^. In addition, the two accompanying satellite peaks are located at approximately 861.00 and 878.87 eV, respectively [20, 22]. The Co 2*p* spectrum of Co_3_O_4_ shows three double peaks at 779.62/794.78, 781.26/796.51 and 786.52/803.17 eV, belonging to Co^3+^, Co^2+^, and satellite peaks, respectively (Fig. 1c) [20, 22]. The binding energy of Ni 2*p* in NiO/Co_3_O_4_ is negatively shifted by 0.28 eV as opposed to NiO, while it is positively shifted by 0.30 eV for Co 2*p* in contrast to Co_3_O_4_, which marks a strong electronic coupling with electrons being transferred from Co_3_O_4_ to NiO. It can be seen that the NiCo MOF precursor (Fig. S4) and NiO/Co_3_O_4_ (Fig. 1d) display nanowire array structures, while NiO shows to the nanosheet structure (Fig. S5). TEM image further confirms that NiO/Co_3_O_4_ has a nanowire structure (Fig. 1e). The HRTEM image of NiO/Co_3_O_4_ shows plane spacing of 0.24 and 0.14 nm, belonging to the (111) plane of NiO and the (440) plane of Co_3_O_4_, respectively (Fig. 1f), with a clear interface between them. In addition, EDS mapping images revealed the presence and uniform distribution of Ni, Co and O elements in the entire nanocomposite material (Fig. 1g). The contents of Co and Ni elements in NiO/Co_3_O_4_ were determined by ICP-OES (Table S1), which shows the catalyst loading on NF is 1.81 mg cm^−2^.

### Electrocatalytic OER and HER Performance

**Fig. 2:**
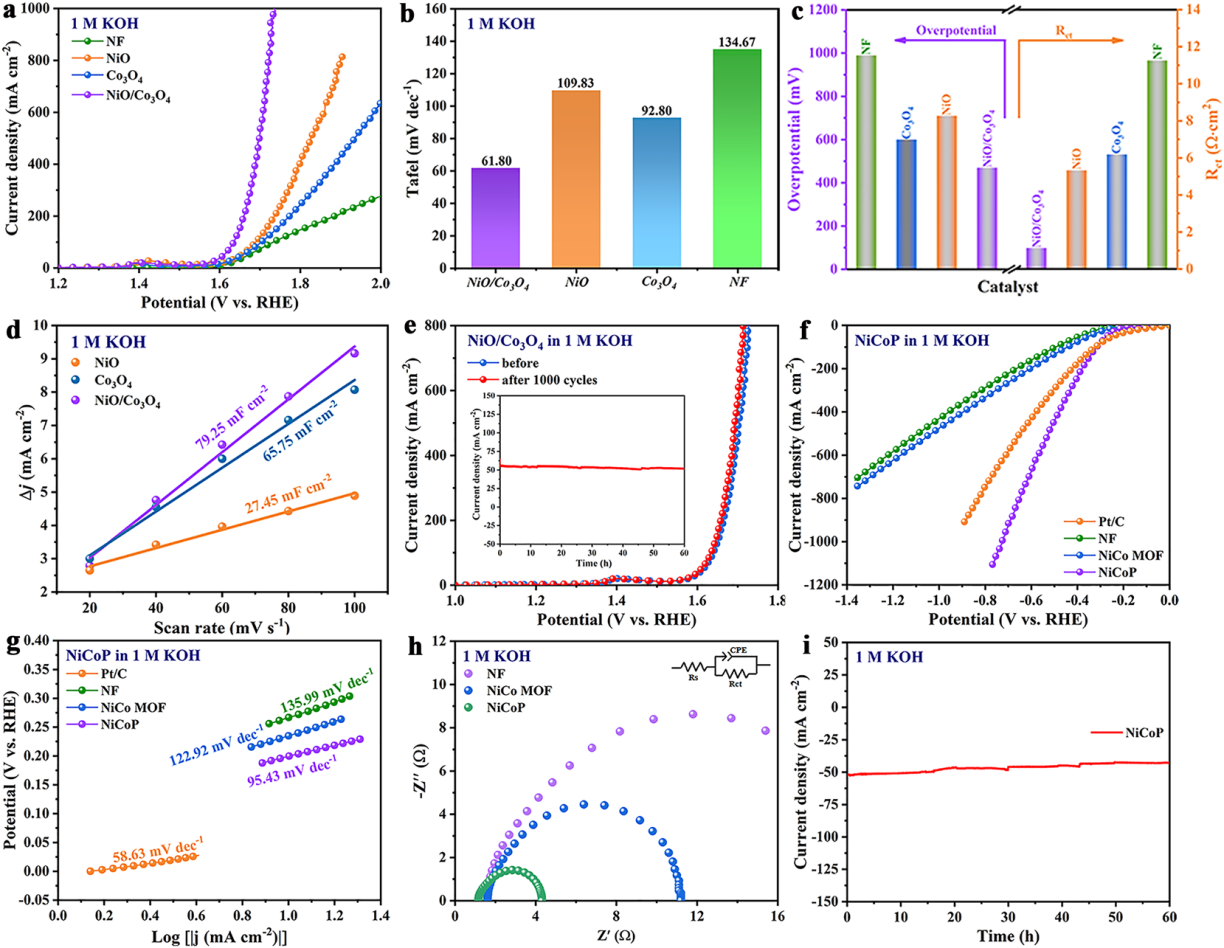
**a** LSV curves, **b** Tafel slopes and **c** overpotentials at 500 mA cm^−2^ and corresponding *R*_ct_ of NF, NiO, Co_3_O_4_ and NiO/Co_3_O_4_ toward OER. **d** Calculated electrochemical C_dl_ for NiO, Co_3_O_4_ and NiO/Co_3_O_4_. **e** LSV curves of NiO/Co_3_O_4_ before and after 1000 CV cycles, and the stability of NiO/Co_3_O_4_ for OER in illustration without *iR* compensation. **f** LSV curves,** g** Tafel slopes of NF, NiCo MOF, NiCoP and Pt/C toward HER. **h** Nyquist plots of different samples for HER. **i** Stability test of NiCoP for HER without *iR* compensation. (Note: All LSV curves in this figure were *iR* compensation)

The OER performance of NF, NiO, Co_3_O_4_ and NiO/Co_3_O_4_ was evaluated in 1 M KOH using a standard three-electrode system. According to LSV curves, NiO/Co_3_O_4_ with a Ni and Co content ratio of 1:3 was selected for further investigation owing to its excellent OER properties (Fig. S6). The LSV curve shows that NiO/Co_3_O_4_ exhibits the best oxygen evolution performance (Fig. 2a), reaching 500 mA cm^−2^ with only a low overpotential of 470 mV, significantly lower than NiO (600 mV), Co_3_O_4_ (710 mV) and NF (990 mV) (Fig. 2c). Tafel slope is obtained by fitting the steady-state LSV curves (Fig. S7), and Tafel slope of NiO/Co_3_O_4_ (61.80 mA dec^−1^) is much lower than that of NF (134.67 mV dec^−1^), NiO (109.83 mV dec^−1^) and Co_3_O_4_ (92.80 mV dec^−1^) (Fig. 2b), implying that NiO/Co_3_O_4_ has excellent reaction kinetics [23, 24]. Charge transfer resistance (R_ct_) was fitted through EIS (Fig. 2c and Table S2), and the smallest R_ct_ value is NiO/Co_3_O_4_ (1.14 Ω), declaring that NiO/Co_3_O_4_ has excellent charge transfer ability [25]. The interface behavior of electron distribution during the OER process can be further explored using in situ EIS [4]. Nyquist plots at different potentials were collected in Fig. S8. As the applied potential increases, the arc of NiO/Co_3_O_4_ significantly decreases compared with NiO and Co_3_O_4_, meaning that the speed of electron transfer is gradually being accelerated. Figure S9 shows the Bode plot of EIS measurements during the OER process. Compared with NiO and Co_3_O_4_, the phase angle of NiO/Co_3_O_4_ in the medium–high-frequency region is less than that in the low-frequency region, which verifies that the electron conduction velocity inside the catalyst is faster compared with the charge transfer velocity at the catalytic interface [26].

ECSA is estimated based on *C*_dl_ obtained from CV curves at different scanning rates [27]. As depicted in Fig. 2d, the C_dl_ value of NiO/Co_3_O_4_ (79.25 mF cm^−2^) is relatively higher compared with that of NiO (27.45 mF cm^−2^) and Co_3_O_4_ (65.75 mF cm^−2^), and the results suggest that NiO/Co_3_O_4_ can expose more catalytic active sites (Fig. S10). The ECSA normalization result shows that NiO/Co_3_O_4_ maintains the best OER performance (Fig. S11). At different potentials, the TOF value of NiO/Co_3_O_4_ is higher compared to NiO and Co_3_O_4_ (Fig. S12). Therefore, NiO/Co_3_O_4_ exhibits excellent intrinsic catalytic activity [28]. In addition to electrochemical activity, the long-term stability of electrocatalysts plays a crucial role in practical applications. Multi-step current and multi-step voltage tests on NiO/Co_3_O_4_ were performed, with the set bypass voltage and current remaining at 300 s (Fig. S13). In Fig. 2e, the LSV curves of NiO/Co_3_O_4_ are almost consistent before and after 1000 cycles. Chronopotentiometric result shows that the NiO/Co_3_O_4_ runs continuously for 60 h without significant attenuation in the current density of 50 mA cm^−2^. After OER stability testing, the nanowire structures of NiO/Co_3_O_4_ show slight changes, and the high-resolution XPS peaks of Ni 2*p* corresponding to Ni^3+^ in NiO/Co_3_O_4_ are significantly increased, showing the occurrence of reconstruction and generation of high-valence species during the OER process (Fig. S14).

In electrocatalysis, traditional TMOs materials are not suitable for HER, because the TMOs undergo their own reduction reaction under the working potential of HER to become pure metals, affecting the stability of TMOs [29, 30]. Herein, NiCo MOF derivatives NiCoP was synthesized for HER using a hydrothermal–phosphating method. As shown in Fig. S15a, the crystal structure of the sample was tested by XRD, and the diffraction peak indexes to NiCoP (PDF#71–2336) [31]. The SEM image shows that NiCoP is in a stacked state, which may be caused by the accumulation of nanowires during phosphating process (Fig. S15b). The HER activity of Pt/C, NF, NiCo MOF and NiCoP in 1 M KOH was evaluated using a standard three-electrode system. The LSV curves of each electrode explain the impressive ultra-low onset potential of NiCoP except for Pt/C (Fig. 2f). Remarkably, NiCoP outperforms Pt/C at current densities greater than 400 mA cm^−2^ and exhibits astonishing performance at high current densities. Tafel slope of NiCoP (95.43 mV dec^−1^) is much smaller than that of NiCo MOF (122.92 mV dec^−1^), NF (135.99 mV dec^−1^), except for Pt/C (58.63 mV dec^−1^), implying that NiCoP has fast HER reaction kinetics (Fig. 2g). The *R*_ct_ values of NiCoP, NiCo MOF and NF are 0.47, 0.91 and 16.46 Ω, respectively, which express NiCoP possesses the lowest charge transfer resistance (Fig. 2h and Table S3). In Fig. S16, NiCoP provides with the maximum *C*_dl_ (3.02 mF cm^−2^), exposing still more active sites. According to results of ECSA normalization (Fig. S17) and TOF (Fig. S18), NiCoP exhibits the best intrinsic catalytic activity. Impressively, even when working continuously for 60 h at a potential of − 1.2 V_RHE_, NiCoP shows no significant change in current density (Fig. 2i). Similarly, the multi-step current and multi-step voltage curves of NiCoP were tested, which maintained at 300 s under different voltages and currents (Fig. S19). The difference in the LSV curve of NiCoP before and after 1000 cycles can be ignored (Fig. S20). After HER testing, there is no significant change in the surface chemical valence state of NiCoP, but the morphological changes are slightly different, possibly due to partial NiCoP dissolution during the HER process (Fig. S21). The P element in the electrolyte of NiCoP after HER electrolysis test was collected and compared with the initial sample, it was found that about 0.005% of P element was leached (Table S4), and the small leaching amount did not affect the stability of the catalyst [32].

### Electrochemical UOR Performance

**Fig. 3:**
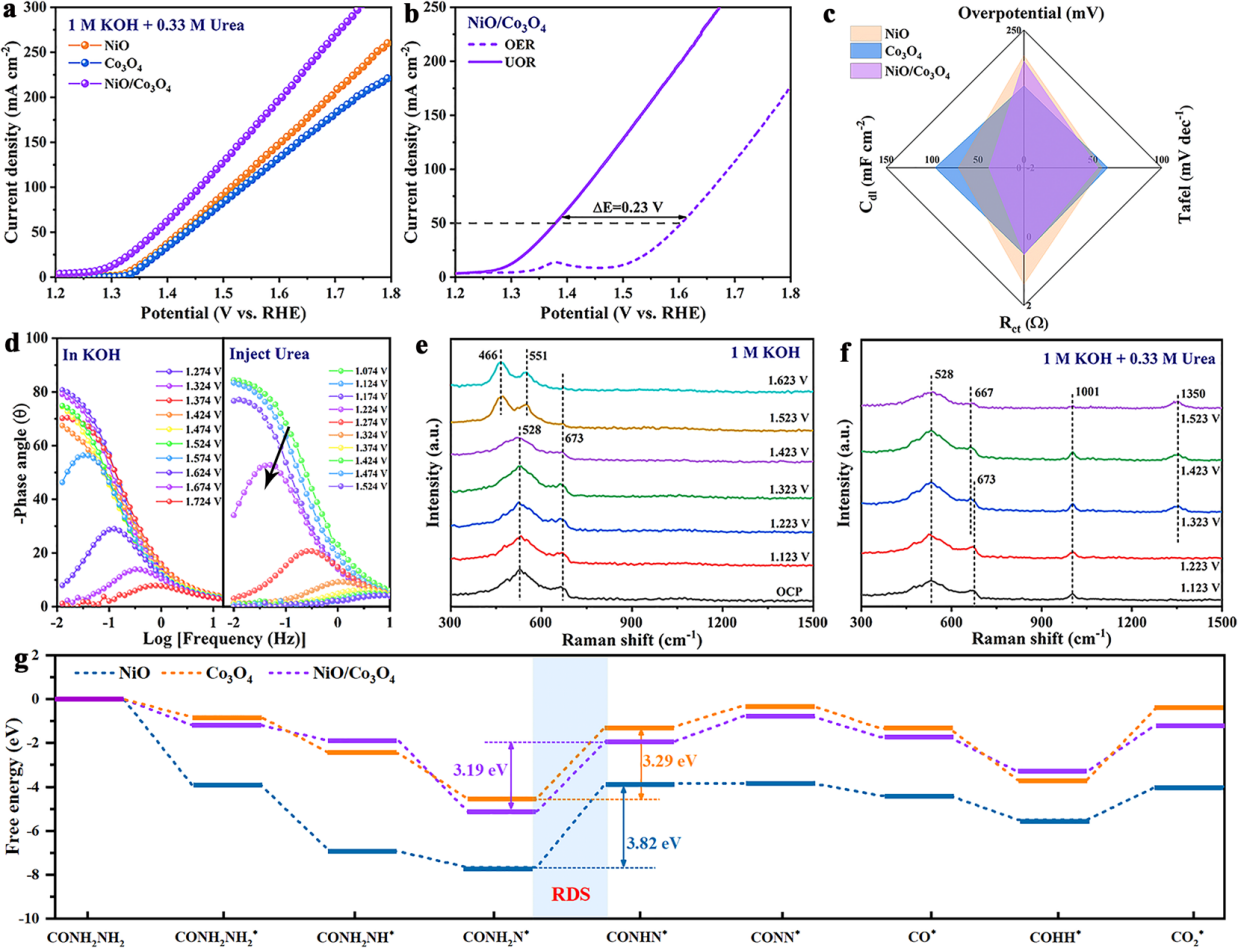
**a** LSV curves of NiO, Co_3_O_4_ and NiO/Co_3_O_4_ toward UOR. **b** Comparison of OER and UOR LSV curves for NiO/Co_3_O_4_. **c** Integrated electrochemical performance radar chart. **d** In situ impedance diagram of NiO/Co_3_O_4_ for OER and UOR. In situ Raman spectra of NiO/Co_3_O_4_ for the **e** OER and **f** UOR processes. **g** Free energy profiles of NiO, Co_3_O_4_ and NiO/Co_3_O_4_ for UOR (Note: All data in this figure were without *iR* compensation)

UOR performances of NiO/Co_3_O_4_, NiO, Co_3_O_4_ and metal phosphides (NiP, CoP and NiCoP) were measured in 1 M KOH containing 0.33 M urea using a standard three-electrode system. During the UOR process, metal phosphides have a low initial potential (Fig. S22). Whereafter, their surface may be oxidized to metal oxides/hydroxides, and the unstable nature of the phosphide causes the UOR performance of NiCoP to be lower than NiO/Co_3_O_4_ when the potential exceeds about 1.42 V_RHE_. As shown in Fig. 3a, b, NiO/Co_3_O_4_ has a lower onset potential in relation to NiO and Co_3_O_4_, superior to most reported oxide catalyst (Table S5), unfolding distinguished UOR performance. Moreover, the corresponding potential of UOR is 1.37 V_RHE_ with the current density at 50 mA cm^−2^, which is negatively shifted by 230 mV compared to OER, showing that UOR is thermodynamically superior to OER. The radar plot shows the combined electrochemical properties of NiO/Co_3_O_4_, NiO and Co_3_O_4_, by comparison, NiO/Co_3_O_4_ exhibits the best electrocatalytic performance for UOR (Figs. 3c, S23, S24 and Table S6).

Generally, a larger decline in the phase angle indicates a faster charge transfer process. For OER, the phase angle of NiO/Co_3_O_4_ in the low-frequency region is stable within the potential range of ~ 1.474 V_RHE_. When the given potential exceeds 1.524 V_RHE_, the phase angle sharply decreases. In contrast, when the UOR potential of NiO/Co_3_O_4_ reached only 1.324 V_RHE_, the phase angle sharply decreased (Fig. 3d). This result demonstrates that NiO/Co_3_O_4_ carries a faster electron transfer rate during UOR process. In Fig. S25, NiO/Co_3_O_4_ was subjected to 6 long-term experiments at a constant potential. (Each experiment lasted for 6 h, and the electrolyte solution was replaced after testing.) At a constant potential of 1.48 V_RHE_, as urea in the electrolyte is gradually consumed, the current density shows a decreasing trend. After replacing the electrolyte, the current density returned to its initial state. Subsequently, the morphology and structure were well maintained, demonstrating excellent structural stability (Fig. S26). The high-resolution XPS spectra of the NiO/Co_3_O_4_ after UOR are shown in Fig. S27. The Ni 2*p* and Co 2*p* deconvoluted into Ni^3+^ and Co^3+^ showed a significant increase compared to the original sample, and the contents of Ni^3+^ and Co^3+^ increased from 60.57% and 38.31% to 69.69% and 45.28%, respectively, which indicate that the surface of NiO/Co_3_O_4_ was partially oxidized and transformed into high-valence species during the UOR process.

Then, in situ Raman spectra are recorded to gain in-depth insight into the electrocatalytic behaviors during UOR catalytic path. When the applied potential is below 1.423 V_RHE_ at 1 M KOH, two distinct Raman peaks appear at 528 and 673 cm^−1^, belonging to NiO and Co_3_O_4_ (Fig. 3e), respectively [33]. Notably, when the applied potential exceeded 1.423 V_RHE_, the peaks of NiO disappeared significantly, and Raman peaks dominated at 466 and 551 cm^−1^ are attributed to the NiOOH species [34]. However, the Raman peak of Co_3_O_4_ did not show any new peaks, indicating that Co_3_O_4_ may not be reconstructed during OER process [35]. In the 1 M KOH with 0.33 M urea, the Raman peak occurring at 1001 cm^−1^ is attributed to urea (Fig. 3f) [36]. When the applied potential exceeded 1.223 V_RHE_, new Raman peaks appeared at 667 and 1350 cm^−1^ belonging to CoOOH and Ni(OH)_2_ [4, 35]. NiO is rapidly converted to NiOOH during the UOR process, and in electrolytes containing nucleophiles (urea), the resulting NiOOH intermediates will be filled with hydrogen of the nucleophile through spontaneous reduction, resulting in their conversion back to Ni(OH)_2_, which is difficult to be recognized by Raman spectroscopy [4, 37]. These results indicate that CoOOH and NiOOH are considered the true active sites of NiO/Co_3_O_4_ in the UOR process. DFT calculations were conducted to further reveal the potential mechanism of the high UOR activity of NiO/Co_3_O_4_ (Fig. S28). The Gibbs free energy diagrams show that the dehydrogenation of the intermediate CONNH_2_^*^ to CONNH^*^ is the rate-determining step (RDS) in the overall UOR process (Fig. 3g) [38]. Evidently, the Gibbs free energy change (ΔG) value for RDS of NiO/Co_3_O_4_ (3.19 eV) is smaller than that of NiO (3.82 eV) and Co_3_O_4_ (3.29 eV), which represents that the NiO/Co_3_O_4_ heterojunction reduces the energy barrier of RDS, thereby increasing the UOR activity [39, 40]. These results further demonstrate the thermodynamic feasibility of promoting UOR by constructing heterogeneous interfaces.

### Electrocatalytic Urea Oxidation Reaction Mechanism

**Fig. 4:**
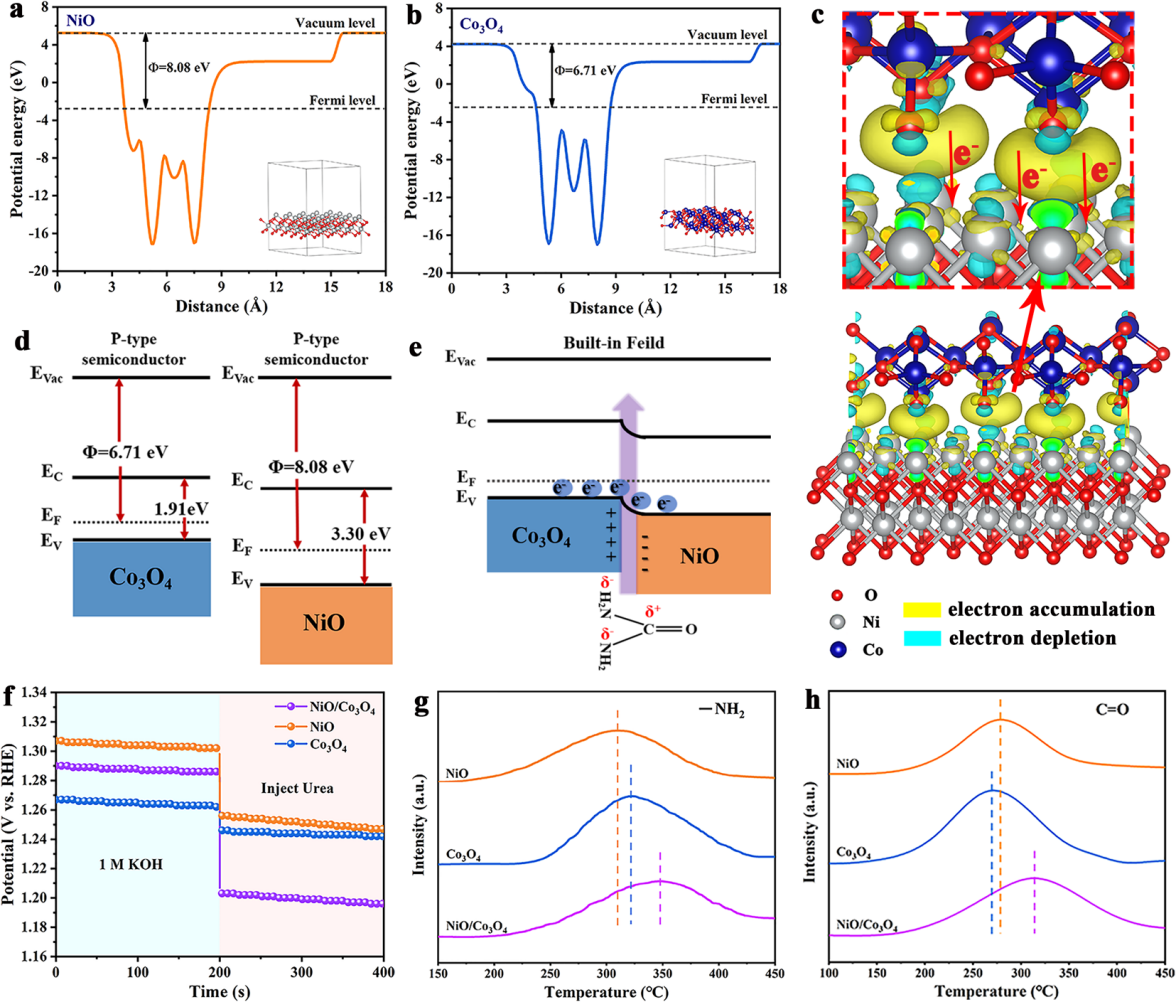
Electrostatic potential of **a** NiO, **b** Co_3_O_4_. **c** Charge density difference in heterostructure of NiO and Co_3_O_4_. Schematic diagrams of the band structure of NiO and Co_3_O_4_ **d** before and **e** after contact. **f** OCP test. TPD adsorption spectra of NiO, Co_3_O_4_ and NiO/Co_3_O_4_ in **g** butylamine/He and **h** CO atmospheres

In electrocatalytic reactions, the surface charge state of catalysts plays a crucial role in the adsorption behavior of reactants [41]. In order to further elucidate the interactions at the heterojunction interface of NiO/Co_3_O_4_, corresponding energy levels were established through Mott–Schottky (M–S) plots, VB-XPS spectrum, UV–Vis diffusive reflectance spectroscopy and work functions. The M–S plots of NiO and Co_3_O_4_ exhibit negative slopes, illustrating that they are p-type semiconductors with flat band potentials (E_FB_) of 1.464 V_RHE_ and 1.224 V_RHE_, respectively (Fig. S29) [42]. Subsequently, the VB-XPS spectra of NiO and Co_3_O_4_ were analyzed, with the valence bands (E_V_) of 1.636 and 1.431 eV (Fig. S30), respectively, which were consistent with the M–S measurement results. The band gaps (E_g_) of NiO and Co_3_O_4_ were obtained by UV–Vis with the values of 3.30 and 1.91 eV, respectively (Fig. S31). DFT calculations indicate that the work functions of NiO and Co_3_O_4_ are 8.08 and 6.71 eV, respectively, meaning that electrons can transfer from Co_3_O_4_ to NiO until the Fermi level reaches equilibrium (Fig. 4a, b). From the analysis of the differential charge density, it can be seen that electrons flow from Co_3_O_4_ to NiO, leading to the depletion electrons on the side Co_3_O_4_, electron aggregation on the NiO side (Fig. 4c). Based on the above results, the energy bands of NiO and Co_3_O_4_, as well as the built-in electric field between them, can be constructed (Fig. 4d, e). When a direct contact is established between NiO and Co_3_O_4_, self-driven electron transfer induces charge redistribution at the interface, resulting in Co_3_O_4_ and NiO becoming electron-deficient and electron-rich regions, respectively.

To verify the effect of heterojunctions on the adsorption behavior of urea, NiO, Co_3_O_4_ and NiO/Co_3_O_4_ were subjected to open circuit potential (OCP) and TPD tests [43, 44]. NiO, Co_3_O_4_ and NiO/Co_3_O_4_ were placed in a solution of 1 M KOH to test their OCP; after 200 s of stabilization, urea (0.33 M) was injected into the solution and stirred evenly (Fig. 4f). The adsorption of organic molecules on the surface of the electrode would lead to the exchange of OH^−^ in the Helmholtz layer of the electrode, resulting in a decrease in the OCP. The larger OCP reduction value of NiO/Co_3_O_4_ indicates that it exhibits stronger adsorption capacity than NiO and Co_3_O_4_ toward urea molecules. Subsequently, the adsorption behavior of NiO, Co_3_O_4_ and NiO/Co_3_O_4_ on urea groups was further studied through TPD testing. In the butylamine/He atmosphere, the desorption temperature for Co_3_O_4_ is higher than that for NiO (Fig. 4g), demonstrating the stronger adsorption of Co_3_O_4_ for the –NH_2_ group. The desorption temperature of NiO for CO exceeds that of Co_3_O_4_, indicating that NiO has a stronger adsorption capacity for C=O groups (Fig. 4h). Surprisingly, the desorption temperature of NiO/Co_3_O_4_ for –NH_2_ and C=O is much higher than that of NiO and Co_3_O_4_; relatively speaking, the synergistic effect of NiO/Co_3_O_4_ heterojunction interface enhances the adsorption capacity of the catalyst for –NH_2_ and C=O.

### AEMWE Performance

**Fig. 5:**
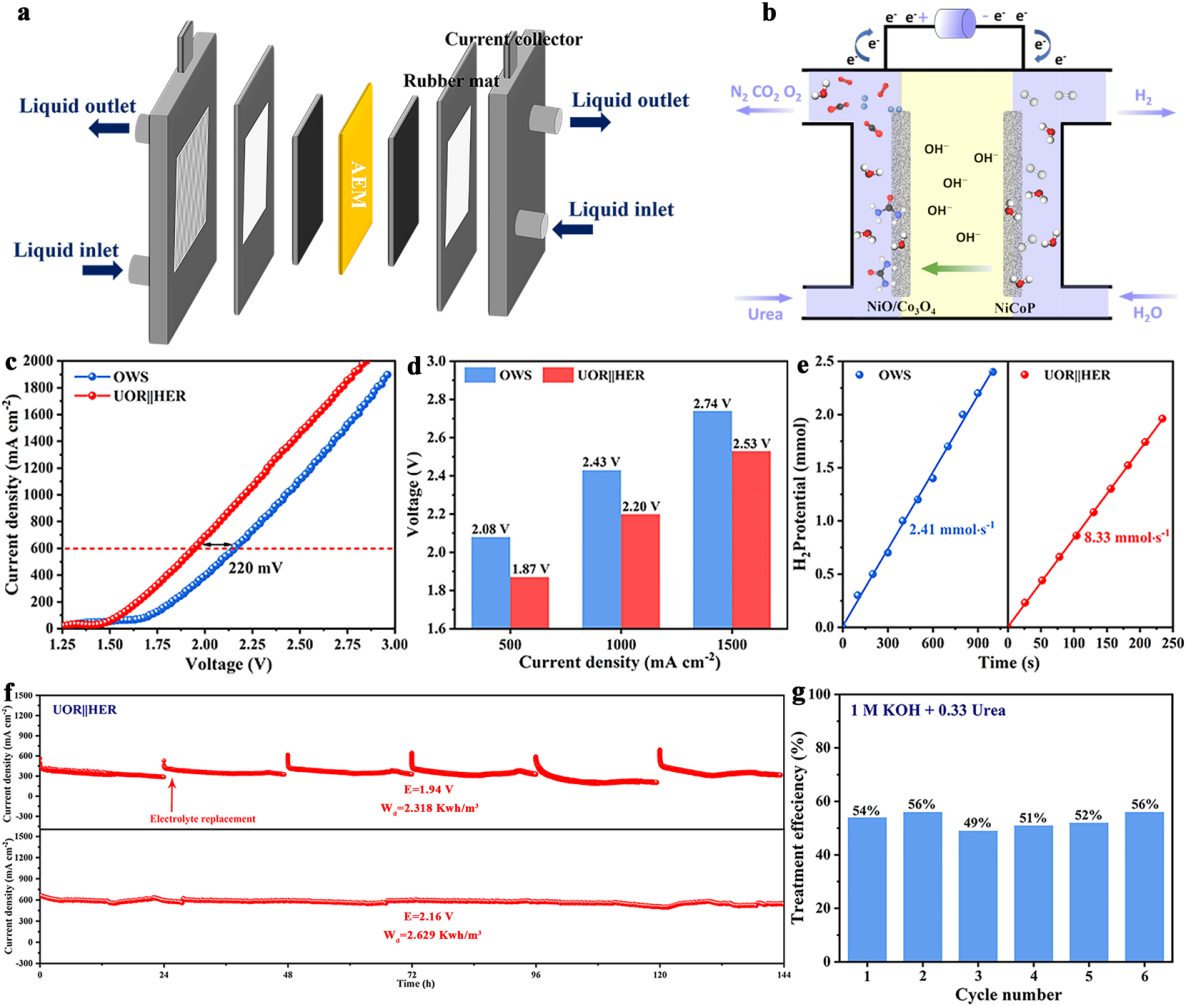
**a** Electrolyzer cell structure model. **b** Two-electrode schematic diagram. **c** LSV curves of NiO/Co_3_O_4_||NiCoP in OWS and UOR||HER. **d** Compare the voltage at different current densities between OWS and UOR||HER. **e** Comparison of H_2_ production rate in OWS and UOR||HER. **f** Long-term stability test for OWS and UOR||HER. **g** Treatment efficiency of urea in UOR||HER system. (Note: All data in this figure were without *iR* compensation)

Considering the excellent electrocatalytic performance of NiO/Co_3_O_4_ in UOR and NiCoP in HER, assembling into AEMWE using NiO/Co_3_O_4_ as anode and NiCoP as cathode (NiO/Co_3_O_4_||NiCoP), its SEUWT coupled hydrogen production performance was studied (Fig. 5a, b) [45]. LSV curves of NiO/Co_3_O_4_||NiCoP during two-electrode electrolysis were compared in different solutions. At a current density of 600 mA cm^−2^, UOR||HER possesses a voltage of 220 mV lower than that of overall water splitting (OWS), indicating that UOR||HER improves the electrolysis efficiency (Fig. 5c). Compared with OWS, UOR||HER saves 210, 230 and 210 mV voltage at 500, 1000 and 1500 mA cm^−2^, reducing overall energy consumption (Fig. 5d). EIS result displays that the reaction kinetic of UOR||HER system is faster, which is more conducive to charge transfer (Fig. S32). Notably, the hydrogen generation rate of UOR||HER reaches 8.33 mmol s^−1^, which is nearly 3.5 times higher than OWS (2.44 mmol s^−1^), implying its distinguished H_2_ generation ability (Fig. 5e). Figure 5f shows that OWS can operate stably for 144 h at 2.16 V with negligible activity decay. In addition, the long-term stability test of UOR||HER was conducted using the chronoamperometric method at a voltage of 1.94 V and 6 consecutive long-term experiments were conducted in the electrolyte. (Each experiment lasted for 24 h, and the electrolyte was replaced after each test.) During this process, each standard cubic meter of H_2_ generates approximately ~ 2.318 kW h of electricity, which is lower than OWS (~ 2.629 kW h) and effectively saves electricity consumption. Due to the continuous consumption of urea during testing, the current density decreased and eventually remained stable, which is due to the severe OER competition caused by the decrease in urea concentration [46]. However, when replacing the electrolyte with fresh urea solution, the required current density will return to its initial state. The improved chemical technology of diacetylacetoxime was used to analyze the concentration of urea-rich wastewater after UOR (Fig. S33). Impressively, the average degradation efficiency of urea in 6 cycles reached 53% (Fig. 5g). Compared with other HER||UOR systems, NiO/Co_3_O_4_||NiCoP electrocatalysts are superior to recently reported works (Table S5). The morphology and structure of NiO/Co_3_O_4_ and NiCoP after UOR show no significant changes (Figs. S34 and S35). The systematic research has verified the impressive stability of NiO/Co_3_O_4_ and NiCoP in the practical application of SEUWT coupled hydrogen production.

## Conclusions

In summary, the NiO/Co_3_O_4_ was synthesized using a hydrothermal–calcination method for SEUWT coupled hydrogen production. DFT calculations indicate that self-driven electron transfer at the NiO/Co_3_O_4_ interface can induce charge redistribution, resulting in NiO and Co_3_O_4_ becoming electron-rich and electron-deficient regions, respectively. Based on TPD and experimental results, it was verified that NiO (electron-rich) and Co_3_O_4_ (electron-deficient) are superior to adsorbed C=O (electron-withdrawing group) and −NH_2_ (electron-donating group), respectively, and the interaction between heterogeneous interfaces regulates the adsorption behavior of the catalyst for urea molecules, accelerating the reaction kinetics of UOR. In AEMWE, the UOR||HER system only requires 1.94 V to reach 600 mA cm^−2^, and H_2_ production increases by about 3.5 times compared to OWS. Meanwhile, the UOR||HER system and OWS system exhibited long-term stability for 144 h at 1.94 and 2.16 V, respectively, and the average urea treatment efficiency in UOR||HER reached 53%. This work is expected to provide broad application prospects in scalable purifying urea-rich wastewater and energy-saving hydrogen production.

## Supplementary Information

Below is the link to the electronic supplementary material.

Supplementary file1 (DOCX 7286 kb)
