# Supplementary material for: Scalable Electrocatalytic Urea Wastewater Treatment Coupled with Hydrogen Production by Regulating Adsorption Behavior of Urea Molecule
Source: Nanomicro Lett. 2025 Feb 24;17:159. doi: 10.1007/s40820-024-01585-0 (PMC11850677; doi:10.1007/s40820-024-01585-0)
Supplement: Supplementary file 1 — Supplementary file1 (DOCX 7286 kb) [file 40820_2024_1585_MOESM1_ESM.docx]

Supporting Information for

**Scalable** **Electrocatalytic Urea Wastewater Treatment** **Coupled with Hydrogen Production by Regulating Adsorption** **Behavior of Urea** **Molecule**

Chunming Yang^1,3,^ *, Huijuan Pang^1^, Xiang Li^1^, Xueyan Zheng^1^, Tingting Wei^1^, Xu Ma^1^, Qi Wang^1^, Chuantao Wang^1,^ *, Danjun Wang^1^ *, Bin Xu^1, 2,^ *

^1^Shaanxi Key Laboratory of Chemical Reaction Engineering, School of Chemistry & Chemical Engineering, Yan’an University, Yan’an 716000, P. R. China

^2^College of Materials Science and Engineering, Beijing University of Chemical Technology, Beijing 100029, P. R. China

^3^Hubei Three Gorges Laboratory, Yichang 443007, P. R. China

*Corresponding authors. E-mail: [chunmingyang@yau.edu.cn](mailto:chunmingyang@yau.edu.cn) (Chunming Yang); [chuantaowang@yau.edu.cn](mailto:chuantaowang@yau.edu.cn) (Chuantao Wang); [wangdj761118@163.com](mailto:wangdj761118@163.com) (Danjun Wang); [xubin@mail.buct.edu.cn](mailto:xubin@mail.buct.edu.cn) (Bin Xu)

**S1 Electrochemical Active Area Normalization**

The formula for calculating the normalized current density of ECSA for the preparation of catalysts is:

ECSA-normalized current density = current density×C_s_ / C_dl_

where C_s_ is the specific capacitance. Usually in alkaline solutions, the value of C_s_ for nickel foam supported catalysts is 0.04 mF cm^-2^. Current density is obtained in the LSV test.

**S2 Conversion frequency (TOF)**

TOF is the total number of molecules catalyzed to the desired product per unit time at each catalytic active site.

$$\mathrm{TOF}=\frac{J}{2n\times F}$$

where *J* is the current density of 1 M KOH hydrogen evolution process, 2 is the stoichiometry of electrons consumed in the HER reaction, n is the number of active sites, and *F* is the Faraday constant (~96485 C mol^-1^).

At 1 M KOH (pH = 13.9), the number of active sites is determined on the CV curve in the potential range at a scanning rate of 100 mV/s. The charge *Q* of the surface-active site (n) can be obtained by the formula *Q = n·F* to describe. Thus, we can calculate the upper limit of the number of active sites (n) according to the following formula:

$$n=\frac{Q}{F}$$

Here, *F* and *Q* correspond to the Faraday constant and the whole charge of CV curve, respectively. Thus, the TOF equation can be calculated as:

$$TOF=\frac{J}{2\frac{Q}{F} x F}=\frac{J}{2Q}$$

*Q* can be calculated according to the following formula:

$$Q=\frac{S}{2v}$$

where *v* is the scan rate and *S* is the integrated area of the CV curve. Finally, the TOF formula is:

$$\text{TOF}\text{=}\frac{\text{Jv}}{\text{S}}$$

**S3 Theoretical Calculations**

All calculations were conducted using the Vienna Computational Simulation Package (VASP) based on DFT. The differential charge density calculations are based on the generalized gradient (GGA) of PBE functional and the basis set of flat wave expansion, with a cutoff energy of 450 eV. The Co_3_O_4_ (440) and NiO (111) were chose to construct a heterojunction composite structure with a lattice mismatch of less than 6%, which basically satisfies the heterojunction requirements with reasonable computational power. The relaxation criterion for the geometric optimization, differential charge density and electrostatic potential were 10-5 eV and 0.02 eV Å^-1^, respectively. To avoid interaction between layers, the vacuum layer in Z direction was set to 15 Å. The Brillouin zone integration was conducted by using a 4×4×1 k-point mesh for structural relaxation and free-energy calculation. The change of Gibbs free energy (ΔG) is defined by:

ΔG = ΔE + ΔZPE – TΔS

here, T denotes temperature and ∆S means the entropy change during the adsorption process, while ∆ZPE is the change of zero-point energies.

**S4 Detection of Urea Concentration**

In this study, the concentration of urea was detected by diacetylacetoxime color method, the principle of which is: urea and diacetyl condensation into red diazine compounds under acidic conditions. Because diacetyl is unstable, it is usually detected with diacetylacetoxime, which first reacts with a strong acid to form diacetyl, and then condensates with urea to form diazine compounds.

The specific operation steps of preparing standard koji by diacetylmonoxime color method are as follows:

Acidic reagent A liquid (10 mL concentrated phosphoric acid + 30 mL concentrated sulfuric acid + 60 mL deionized water + 10 mg ferric chloride), diacetylmonoxime reagent B liquid (0.5 g diacetylmonoxime + 10 mg thiosemicarbazone + 100 mL deionized water), respectively. Solution with urea concentration of 0, 1, 2, 5, and 10 ppm were configured. After mixing 2 mL A liquid, 1 mL B liquid and 1mL urea solution evenly, the water bath was heated to 100 ℃ and kept for 15 min. After the solution was cooled to room temperature, the absorbance was tested at the wavelength of 525 nm. The absorbance standard curve of urea at 525 nm was obtained by linear fitting of different concentrations of urea solution.

**S5 Temperature-programmed Desorption (TPD) Testing Details and Process**

TPD testing method for -NH_2_: The -NH_2_ of carbonyl on the active solid component was determined by TPD of NH_3_. The adsorption instrument adopted the chemisorption 2920II of the American MAC Company. The sample after reaction was weighed at 0.1 g and put into a quartz tube, which was passed into high purity Ar and then increased to 200 ℃ at a rate of 10 ℃/min. After 2 h of purging, the temperature was lowered to 50 ℃, and NH_3_ was added for adsorption. After 3 h of adsorption, the temperature was raised to 200 ℃, and the physical adsorption of NH_3_ was removed by purging, followed by Ar purging for 30 min, and then rising to 800 ℃ at a rate of 10 ℃/min.

TPD testing method for C=O: The chemisorption of C=O on the active solid component was determined by TPD of CO_2_. The adsorption instrument adopted the chemisorption 2920II of the American MAC Company. The sample after reaction was weighed at 0.1 g and put into a quartz tube, which was passed into high purity Ar and then increased to 200 ℃ at a rate of 10 ℃/min. After 2 h of purging, the temperature was lowered to 50 ℃, and CO_2_ was added for adsorption. After 3 h of adsorption, the temperature was raised to 200 ℃, and the physical adsorption of CO_2_ was removed by purging, followed by Ar purging for 30 min, and then rising to 800 ℃ at a rate of 10 ℃/min.

**Supplementary Figures and Tables**





**Fig. S1** The LSV performance of different MOF precursors and their oxide (All LSV curves were *iR* compensation)


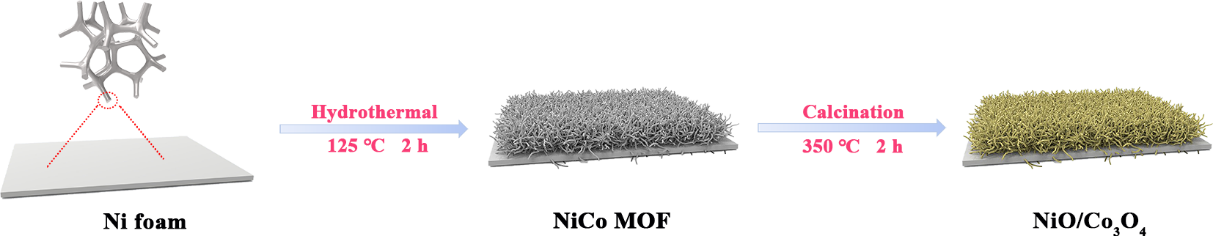


**Fig. S2** Schematic diagram of NiO/Co_3_O_4_ synthesis


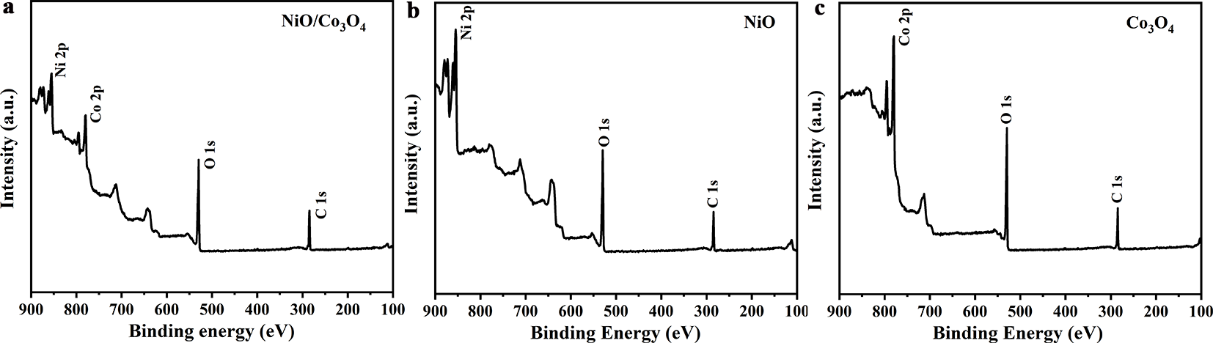


**Fig. S3** XPS survey spectrums of NiO/Co_3_O_4_, NiO and Co_3_O_4_


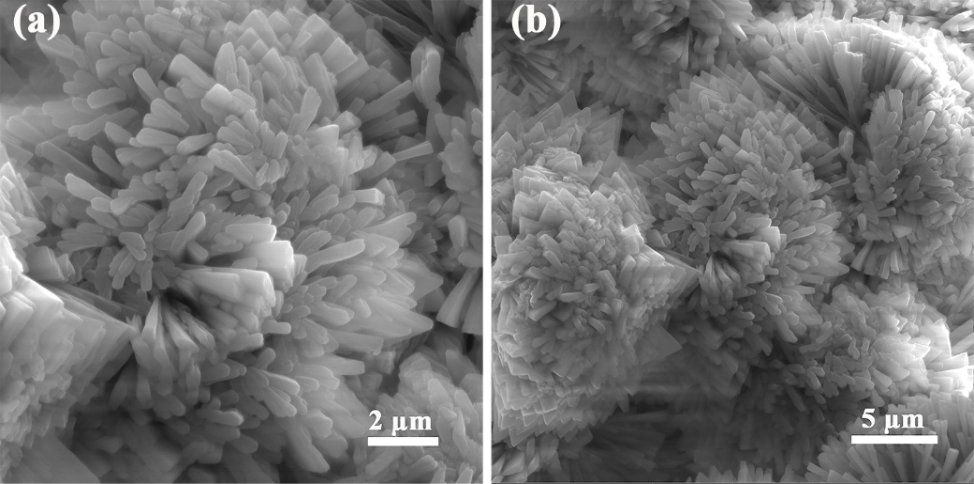


**2 µm**

**Fig. S4** SEM images of the NiCo MOF precursor


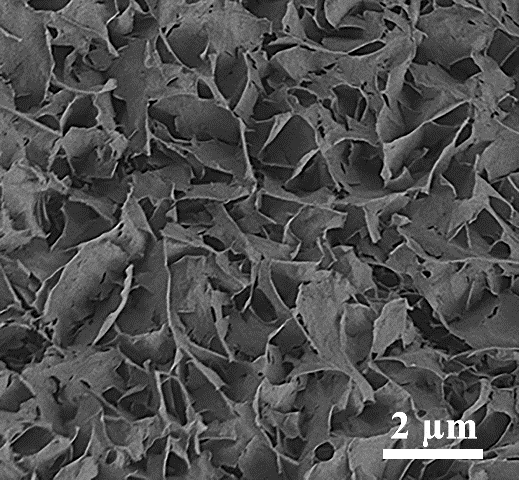


**Fig. S5** SEM image of the NiO





**Fig. S6** Comparison of the LSV curves of Ni:Co with different proportions in NiO/Co_3_O_4_


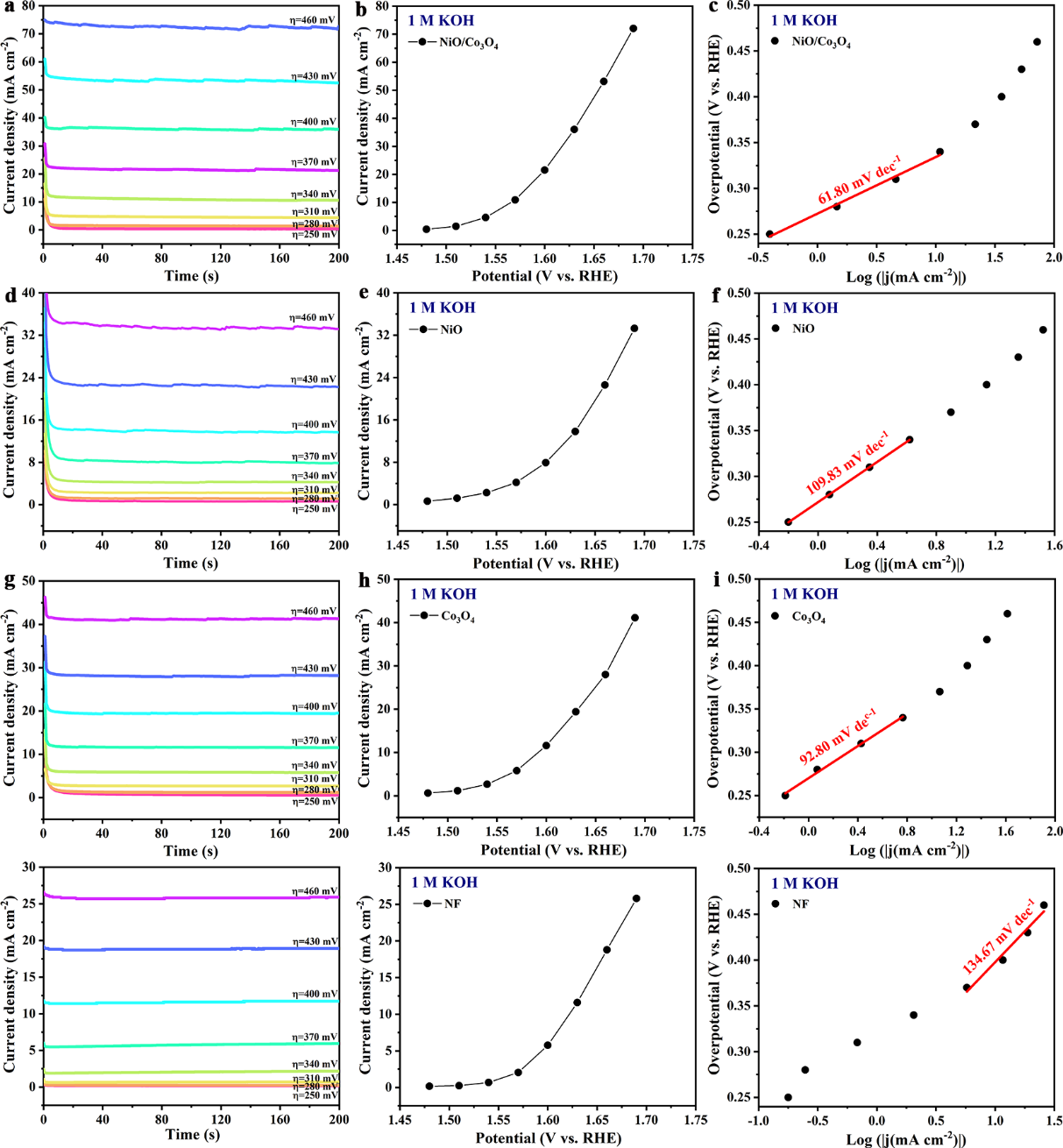


**Fig. S7** Chronoamperometry (CA) responses, the plot of OER current densities and Tafel plot constructed from OER current densities from steady-state CA responses with iR of NF, NiO, Co_3_O_4_, and NiO/Co_3_O_4_ catalysts in 1 M KOH solution


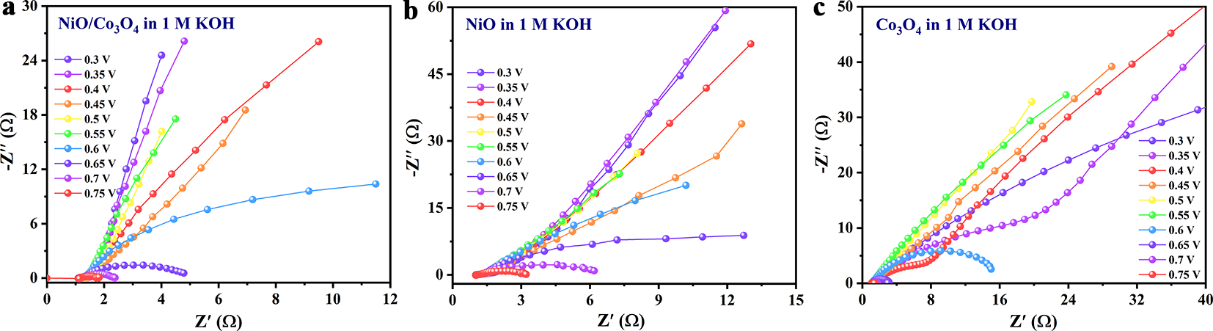


**Fig. S8** Nyquist plots for (**a**) NiO/Co_3_O_4_, (**b**) NiO and (**c**) Co_3_O_4_ at different applied potentials in 1 M KOH solution


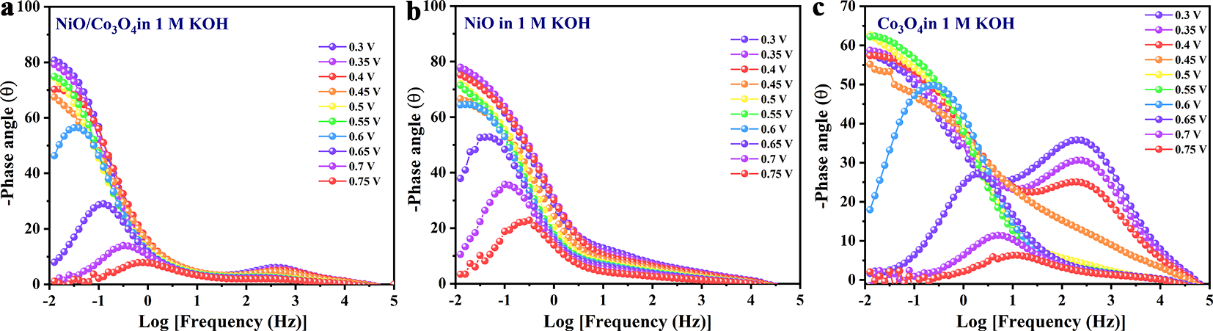


**Fig. S9** Nyquist plots from 100 kHz to 0.01 kHz. Bode plots of (**a**) NiO/Co_3_O_4_, (**b**) NiO and (**c**) Co_3_O_4_


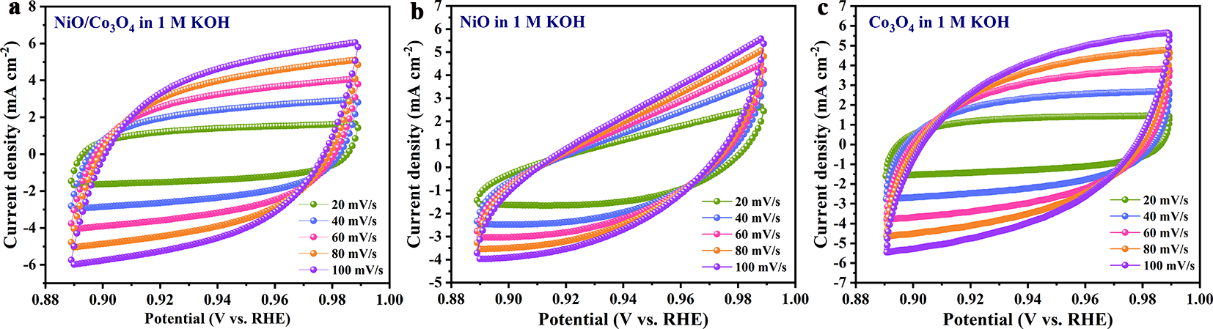


**Fig. S10** CV curves of (**a**) NiO/Co_3_O_4_, (**b**) NiO, (**c**) Co_3_O_4_ for OER in capacitive region at different scan rates





**Fig. S11** ECSA-normalized LSV curves of NiO/Co_3_O_4_, NiO and Co_3_O_4_ in 1 M KOH





**Fig. S12** Comparison of the TOF values of NiO/Co_3_O_4_, NiO and Co_3_O_4_ for OER


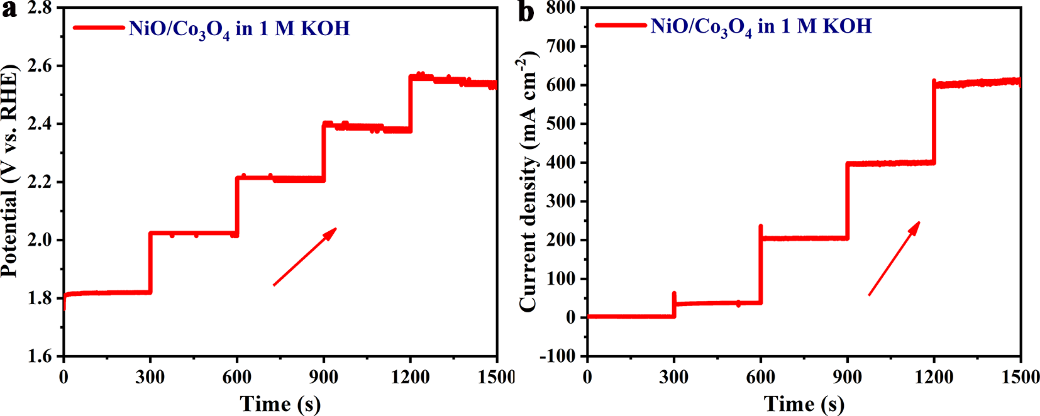


**Fig. S13** Multi-current process and multi-potential process of NiO/Co_3_O_4_ in 1 M KOH (without *iR* compensation)


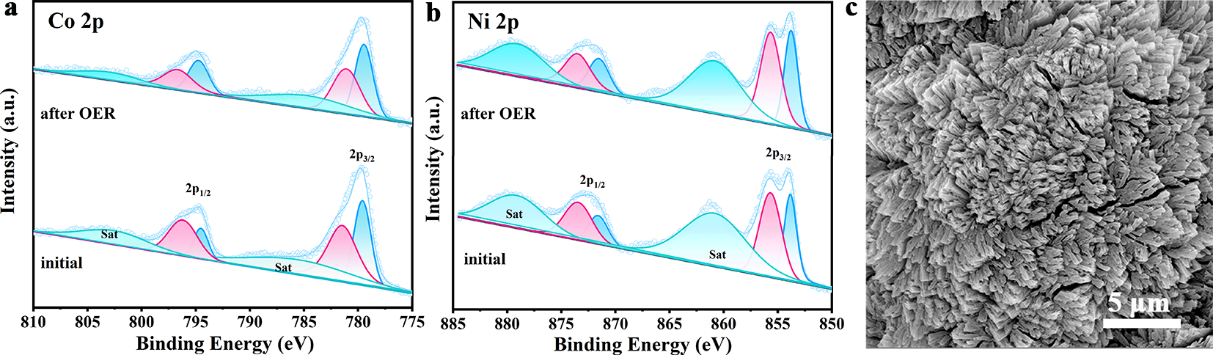


**Fig. S14** High-resolution XPS spectrums of (**a**) Ni 2p, (**b**) Co 2p before and after OER. (**c**) SEM image of the NiO/Co_3_O_4_ before and after OER


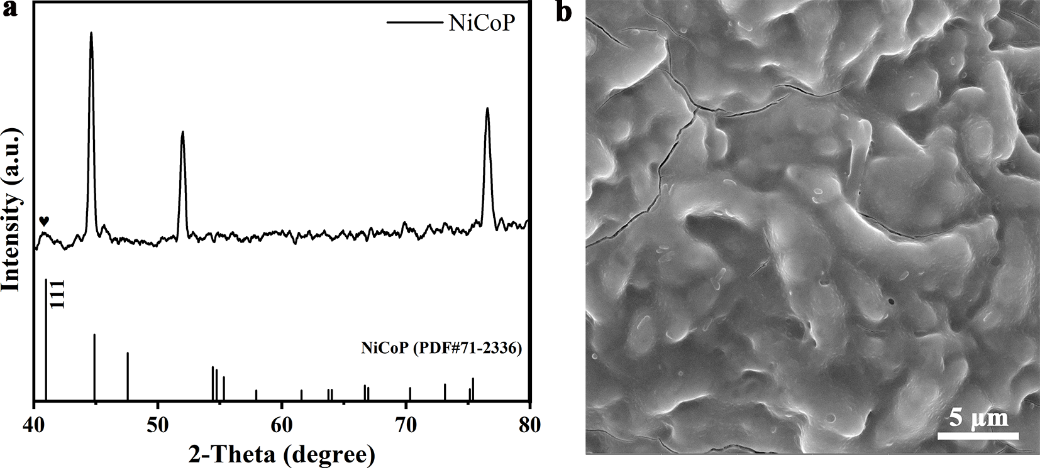


**Fig. S15** (**a**) XRD pattern, (**b**) SEM image of NiCoP


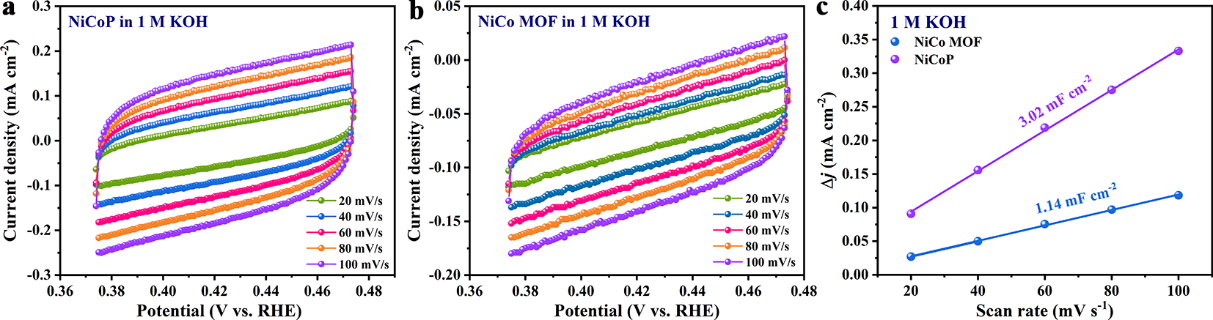


**Fig. S16** CV curves of (**a**) NiCoP, (**b**) NiCo MOF for HER in capacitive region at different scan rates





**Fig. S17** ECSA-normalized LSV curves of NiCoP, NiCo MOF in 1 M KOH





**Fig. S18** Comparison of the TOF values of NiCoP, NiCo MOF for HER


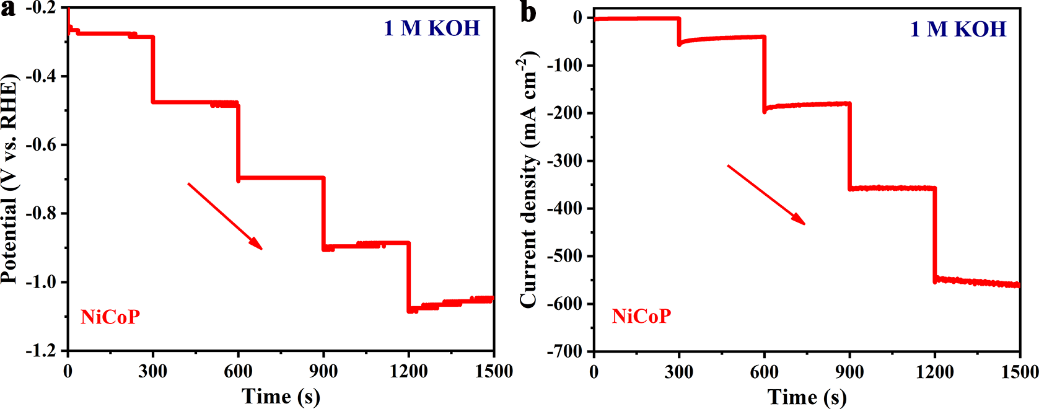


**Fig. S19** Multi-current process and multi-potential process of NiCoP in 1 M KOH (without *iR* compensation)





**Fig. S20** LSV curves before and after 1000 circulation


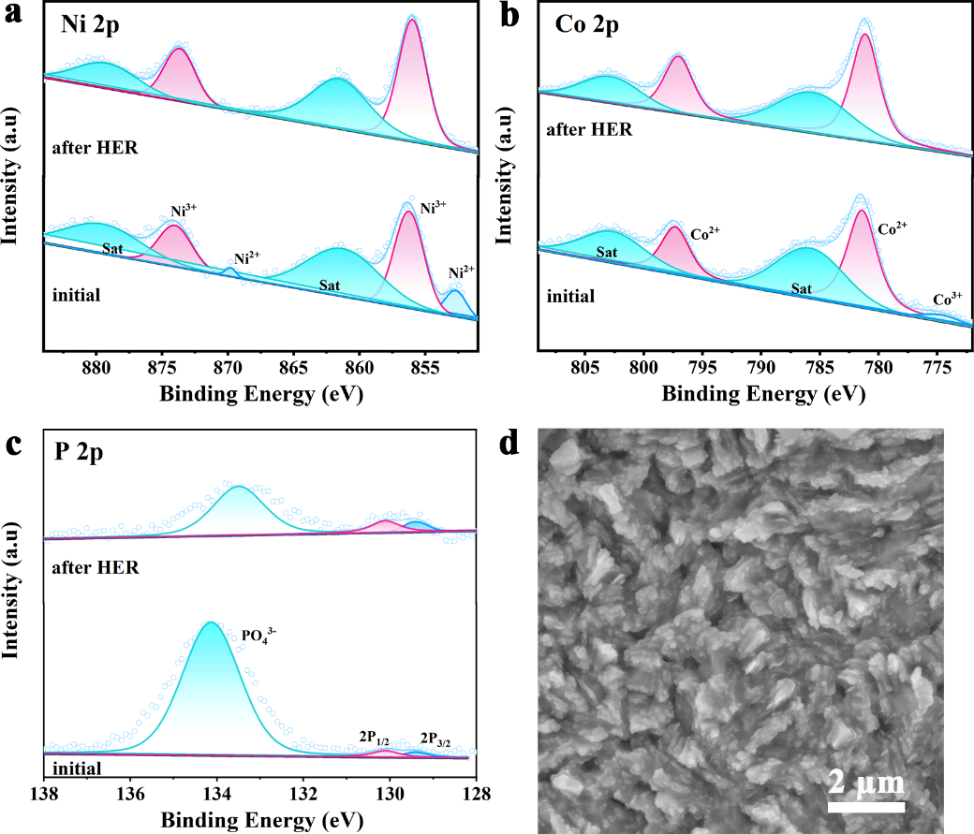


**Fig. S21** (**a**) High-resolution XPS spectrums of Ni 2p, (**b**) Co 2p and (**c**) P 2p before and after HER. (**d**) SEM image of the NiCoP after HER





**Fig. S22** LSV curves of NiO, Co_3_O_4_, NiO/Co_3_O_4_, NiP, CoP, and NiCoP toward UOR. (Note: All LSV curves were without *iR* compensation)


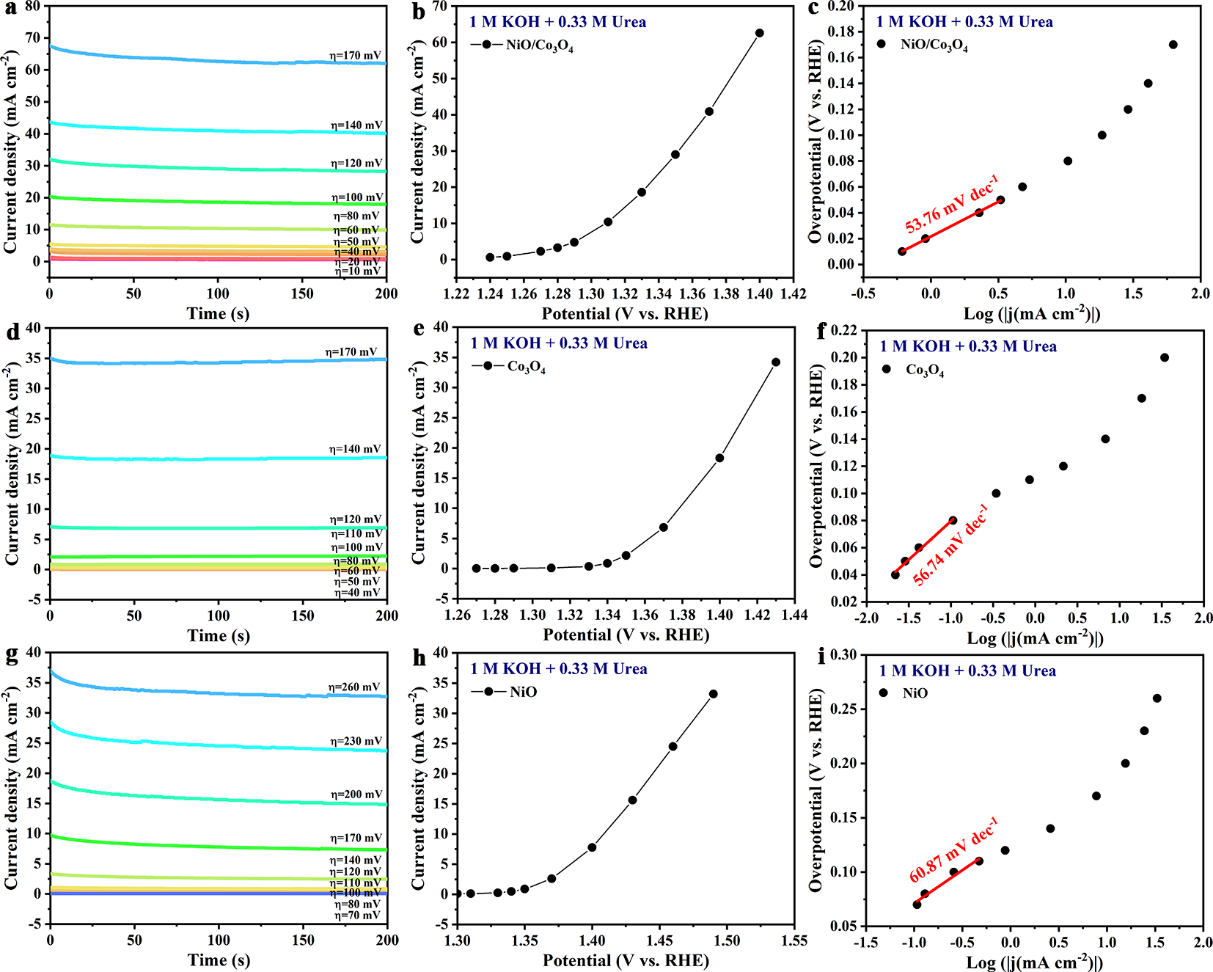


**Fig. S23** Chronoamperometry (CA) responses, the plot of UOR current densities and Tafel plot constructed from UOR current densities from steady-state CA responses with iR of NF, NiO, Co_3_O_4_, and NiO/Co_3_O_4_ catalysts in 0.33 M urea solution


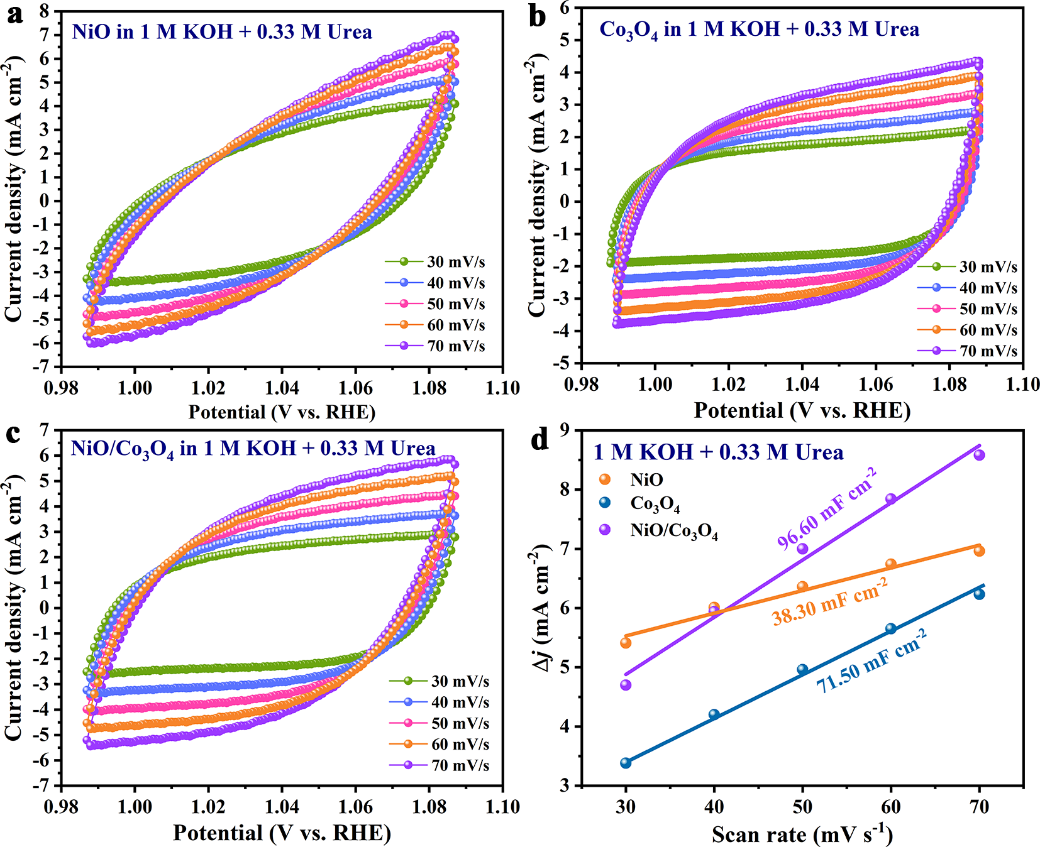


**Fig. S24** CV curves of (**a**) NiO/Co_3_O_4_, (**b**) NiO and (**c**) Co_3_O_4_ for UOR in capacitive region at different scan rates. (**d**) Calculated electrochemical C_dl_ for NiO, Co_3_O_4_, NiO/Co_3_O_4_ for UOR


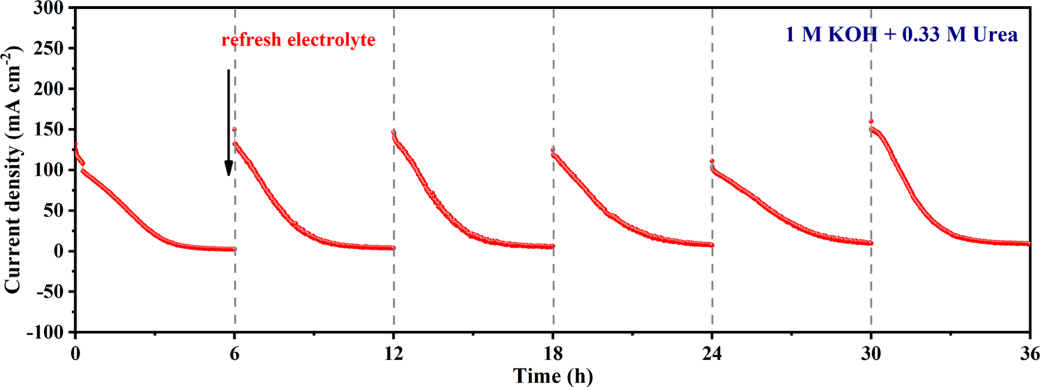


**Fig. S25** Stability cycle test for 36 h


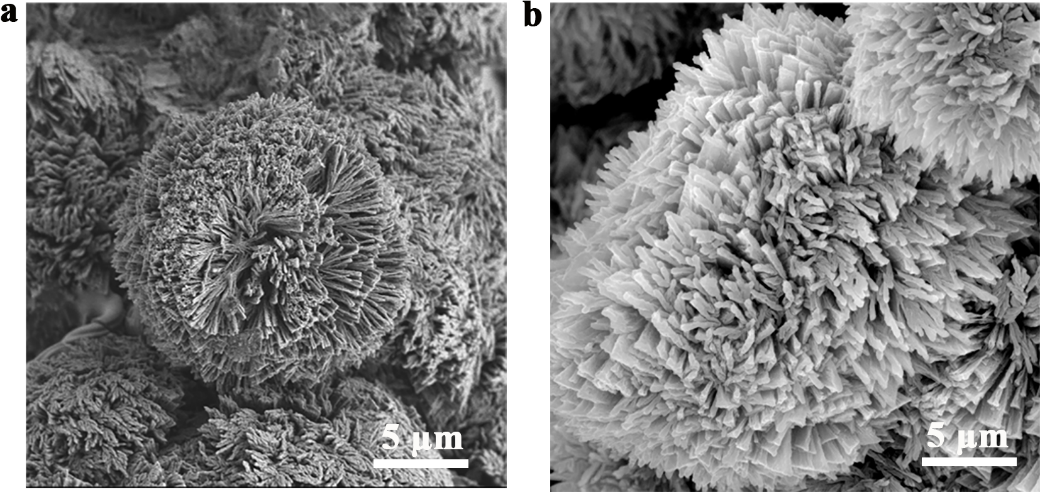


**Fig. S26** SEM images of the NiO/Co_3_O_4_ (**a**) before and (**b**) after UOR


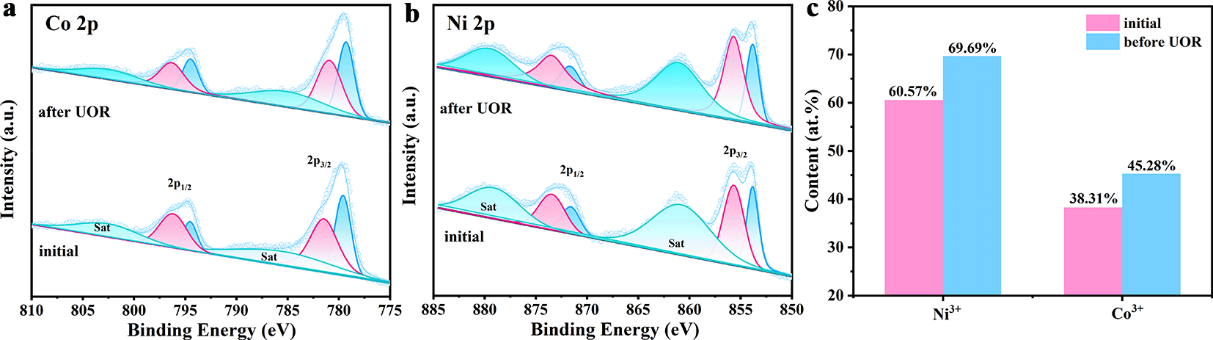


**Fig. S27** High-resolution XPS spectrums of (**a**) Co 2p, (**b**) Ni 2p before and after UOR. (**c**) comparison of the content for Ni^3+^, Co^2+^ calculated by XPS results of NiO/Co_3_O_4_ before and after UOR


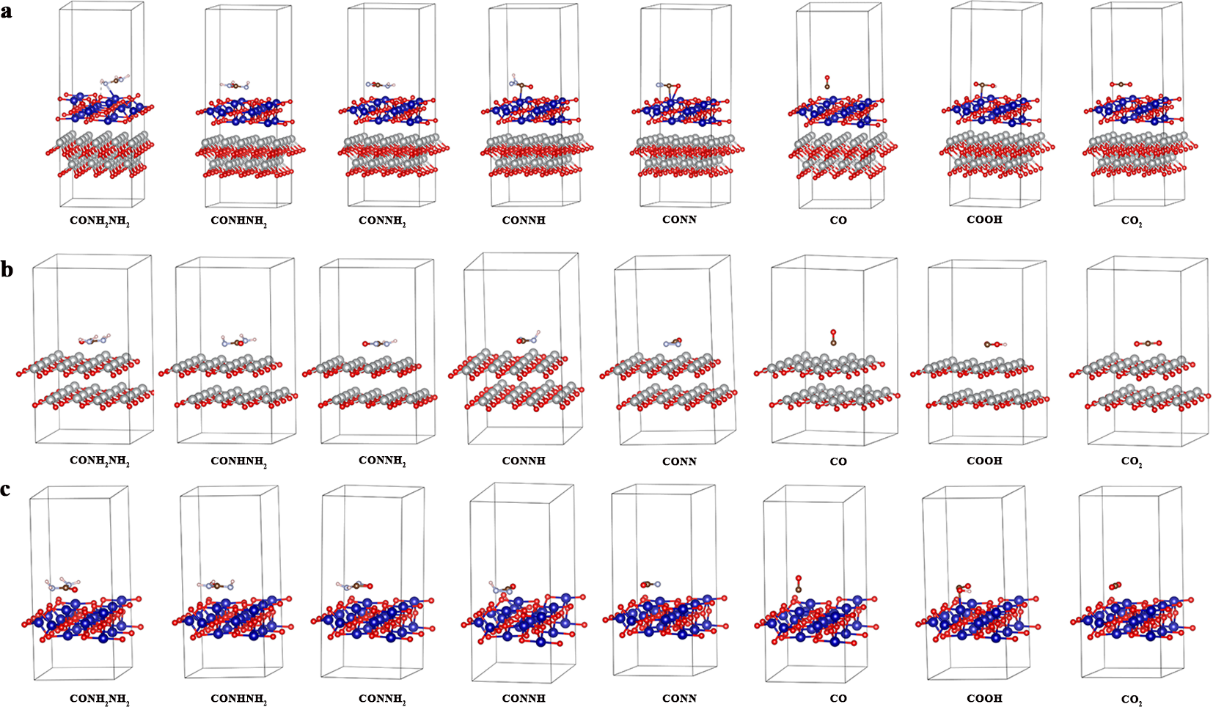


**Fig. S28** Model structures of intermediates on the surface of (**a**) NiO/Co_3_O_4_, (**b**) NiO and (**c**) Co_3_O_4_ during UOR process


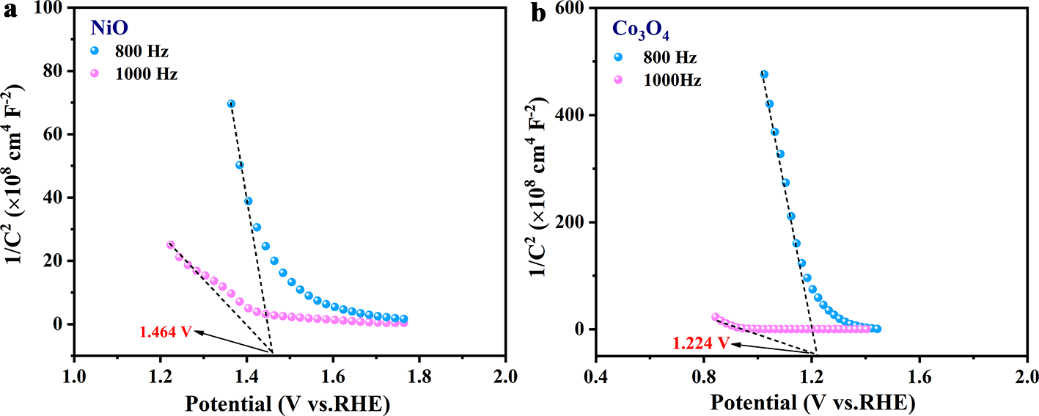


**2 µm**

**Fig. S29** Mott-Schottky plots of (**a**) NiO, (**b**) Co_3_O_4_


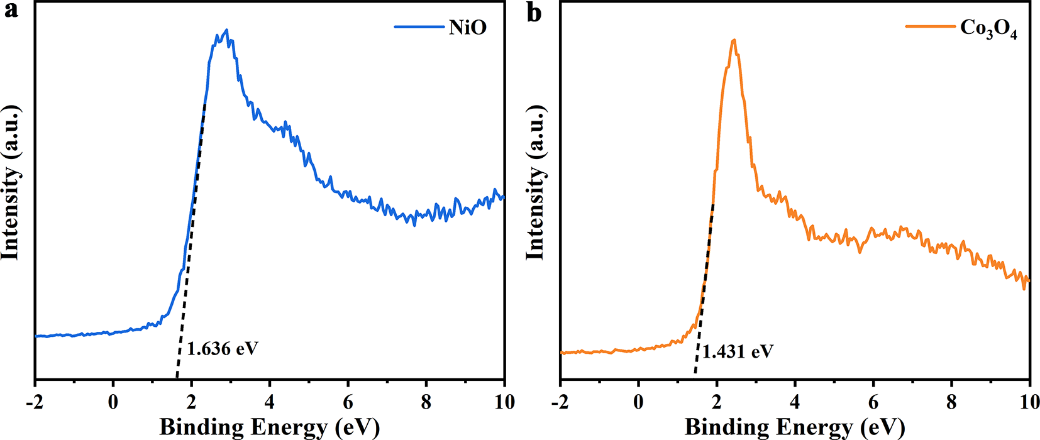


**Fig. S30** VB-XPS spectrums of (**a**) NiO, (**b**) Co_3_O_4_

**
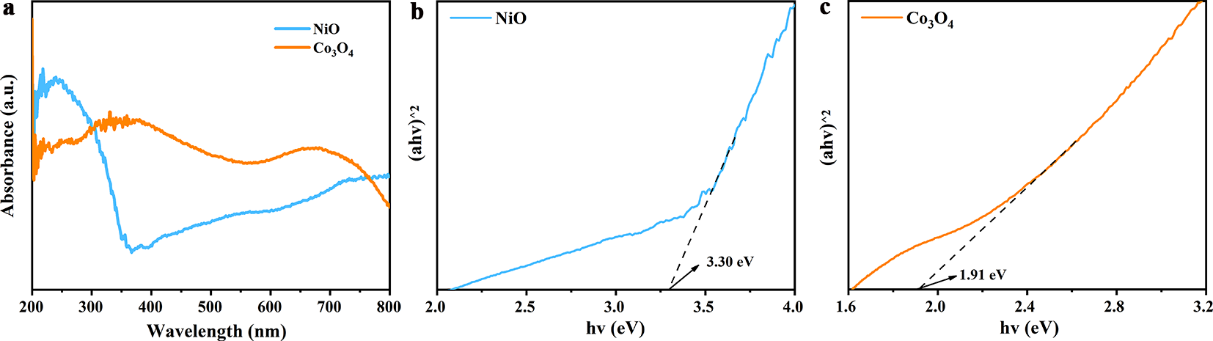
**

**Fig. S31** (**a**) UV–vis spectrums of NiO, and Co_3_O_4_. Corresponding the Tauc plots of (**b**) NiO and (**c**) Co_3_O_4_





**Fig. S32** Nyquist plot derived from EIS of OWS and UOR||HER, respectively


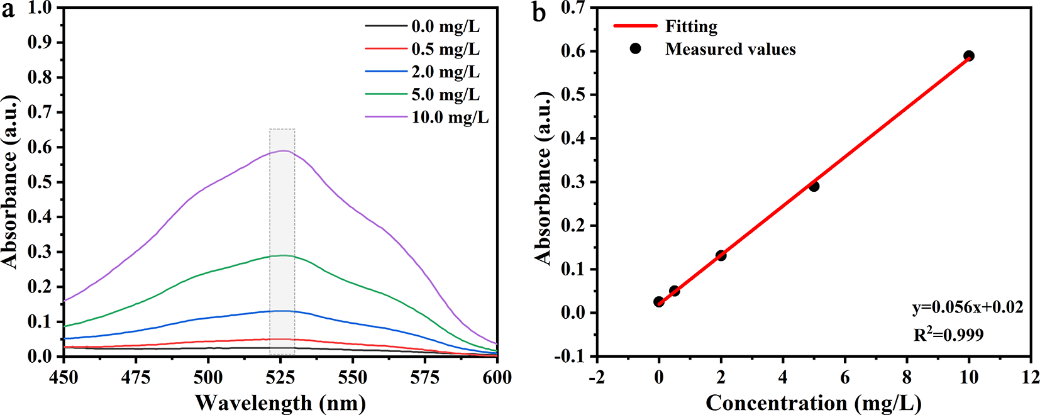


**Fig. S33** Proved standard curve for the determination of urea solutions at different concentrations by diacetylacetoxime chemistry

**2 µm**

**2 µm**


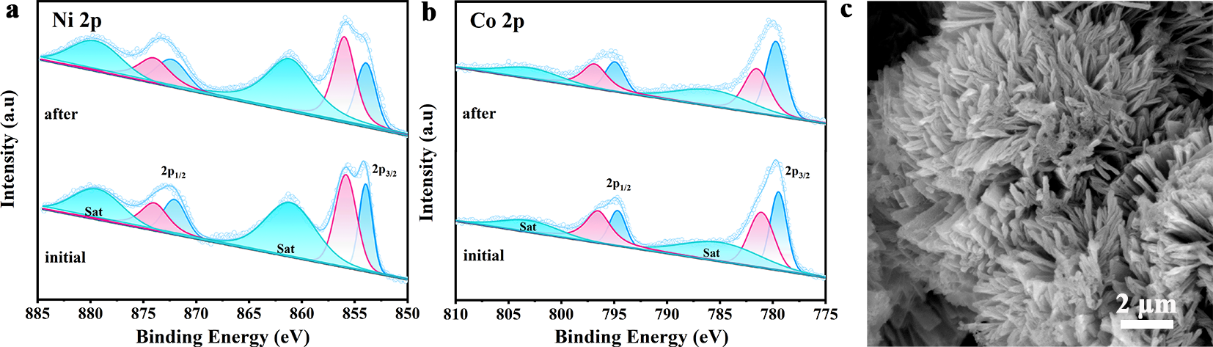


**Fig. S34** (**a**) High-resolution XPS spectrums of Ni 2p, (**b**) Co 2p before and after UOR||HER. (**c**) SEM image of the NiO/Co_3_O_4_ after UOR||HER


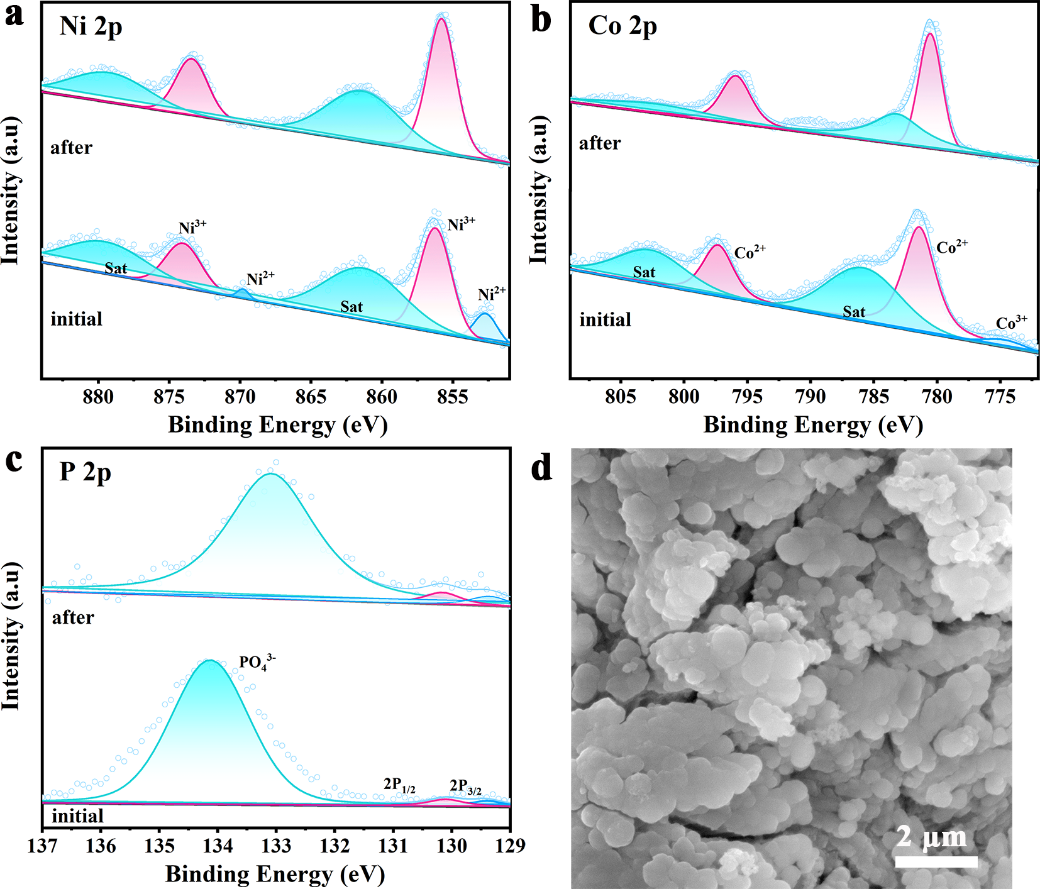


**Fig. S35** High-resolution XPS spectrums of (**a**) Ni 2p, (**b**) Co 2p and (**c**) P 2p before and after UOR||HER. (**d**) SEM image of the NiCoP after UOR||HER

**Table S1** Contents of Co, Ni in NiO/Co_3_O_4_

| Catalyst | Element | Weight ratio of element (%) | Catalyst loading capacity/mg cm^-2^ |
| --- | --- | --- | --- |
| NiO/Co_3_O_4_ | Co | 68.91 | 1.81 |
|  | Ni | 31.09 |  |
|  | O | / |  |

**Table S2** R_s_, CPE and R_ct_ of electrocatalysis in 1 M KOH for OER

| Catalyst | R_s_ (Ω) | CPE | R_ct_ (Ω) |
| --- | --- | --- | --- |
| NiO/Co_3_O_4_ | 0.7178 | 0.6587 | 1.136 |
| NiO | 0.9869 | 0.5650 | 5.337 |
| Co_3_O_4_ | 1.003 | 0.7422×10^-1^ | 6.192 |
| NF | 1.152 | 0.2412×10^-2^ | 1.26 |

**Table S3** R_s_, CPE and R_ct_ of electrocatalysis in 1 M KOH for HER

| Catalyst | R_s_ (Ω) | CPE | R_ct_ (Ω) |
| --- | --- | --- | --- |
| NiCoP | 1.161 | 0.2284×10^-2^ | 0.4745 |
| NiCo MOF | 1.607 | 0.3330×10^-2^ | 0.9068 |
| NF | 1.6180 | 0.5823×10^-2^ | 16.46 |

**Table S4** The concentrations of P element in the solution of NiCoP after HER reactions

| Simple quality/g | Constant volume/mL | Dilution factor | Element | Instrument  readings/mg L^-1^ | Sample element content (%) |
| --- | --- | --- | --- | --- | --- |
| / | / | / | P | 4.98 | 0.0050 |
| 0.1472 | 100 | 10 | P | 5.6116 | 3.8122 |

**Table S5** Comparison of UOR performance of NiO/Co_3_O_4_ with the reported oxide catalysts in 1.0 M KOH solution

| Catalyst | Urea concentration  in 1 M KOH | Potential (V_RHE_) for the UOR at certain current density (mA cm^-2^) | DOI |
| --- | --- | --- | --- |
| **NiO/Co_3_O_4_** | **0.33** | **1.37(50)/1.32(10)** | **This work** |
| FeOOH@Co_3_O_4_ | 0.33 | 1.43(10) | 10.1016/j.jcis.2022.05.070 |
| NiO-CrO_x_ | 0.33 | 1.37(50) | 10.1007/s12274-022-4635-5 |
| Ni/MNO-10 | 0.5 | 1.37(10) | 10.1002/sstr.202300212 |
| NiO-NiPi/NF | 0.5 | 1.35(10) | 10.1016/j.cej.2021.130514 |
| NiMoO4•xH_2_O | 0.33 | 1.41(10) | 10.1016/j.electacta.2014.11.193 |
| Ce-Co_3_O_4_ | 0.5 | 1.39 (50) | 10.1016/j.jcis.2022.10.031 |
| CoMoO@Co/GF | 0.5 | 1.35 (20) | 10.1016/j.jcis.2021.12.149 |
| NiSe_2_-NiO | 0.33 | 1.33(10) | 10.1016/j.apcatb.2020.119165 |
| (FeNiCoCrCu)_3_O_4_ | 0.33 | 1.35(10) | 10.1021/acsami.2c09161 |
| H_2_-NiFe/Co_3_O_4_ | 0.33 | 1.32(10) | 10.1021/acs.iecr.2c02958 |

**Table S6** R_s_, CPE and R_ct_ of electrocatalysis in 1 M KOH with 0.33 M urea

| Catalyst | R_s_ (Ω) | CPE | R_ct_ (Ω) |
| --- | --- | --- | --- |
| NiO/Co_3_O_4_ | 0.9048 | 0.3572 | 0.5062 |
| NiO | 1.025 | 0.1247 | 0.5365 |
| Co_3_O_4_ | 1.033 | 0.7301×10^-1^ | 1.387 |

**Table S7** Comparison of the urea-assisted hydrogen evolution reaction performance of NiO/Co_3_O_4_||NiCoP with the reported catalysts

| Catalysts | Urea concentration  in 1 M KOH | Potential (V) for the HER\|\|UOR at certain current density (mA cm^-2^) | Stability  (h/mA cm^-2^) | DOI |
| --- | --- | --- | --- | --- |
| **NiO/Co_3_O_4_(+)\|\|NiCoP(-)** | **0.33** | **1.15(10)/1.49(50)/1.55(100)** | **144/600** | **This work** |
| Fe-Co_0.85_Se/FeCo LDH | 0.5 | 1.32(10) | 60/100 | 10.1002/adfm.202212811 |
| Ni/MNO-10(+)\|\|Pt/C(-) | 0.5 | 1.45(10) | 12/10 | 10.1002/sstr.202300212 |
| CrCoNiFe(+)\|\|Pt(-) | 0.33 | 1.83(50) | 60/20 | 10.1016/j.jcis.2023.04.055 |
| V-Co_2_P_4_O_12_/CC | 0.5 | 1.42(10) | 20/10 | 10.1002/adfm.202313974 |
| Ni_2_P_4_O_12_/NiTe | 0.33 | 1.37(10) | 500/100 | 10.1002/adma.202311766 |
| RhSA-S-Co_3_O_4_ | 0.5 | 1.33(10) | 120/100 | 10.1039/D1EE02603H |
| Ni_3_N/Mo_2_N | 0.33 | 1.36(10)/1.47(50) | 50/10 | 10.1021/acscatal.3c00113 |
| Mn-Ni(OH)_2_/CP | 0.5 | 1.41(10) | 12/10 | 10.1021/acsestengg.1c00400 |
| NiF_3_/Ni_2_P@CC-2 | 0.33 | 1.54(10) | 10/10 | 10.1016/j.cej.2021.130865 |
